# Recasting Current Knowledge of Human Fetal Circulation: The Importance of Computational Models

**DOI:** 10.3390/jcdd10060240

**Published:** 2023-05-30

**Authors:** Daibo Zhang, Stephanie E. Lindsey

**Affiliations:** Department of Mechanical and Aerospace Engineering, University of California, San Diego, CA 92093, USA; daz045@eng.ucsd.edu

**Keywords:** fetal circulation, pediatric cardiology, congenital heart defects, growth restriction, hemodynamics, computational fluid dynamics, cardiovascular lumped-parameter networks, patient-specific modeling

## Abstract

Computational hemodynamic simulations are becoming increasingly important for cardiovascular research and clinical practice, yet incorporating numerical simulations of human fetal circulation is relatively underutilized and underdeveloped. The fetus possesses unique vascular shunts to appropriately distribute oxygen and nutrients acquired from the placenta, adding complexity and adaptability to blood flow patterns within the fetal vascular network. Perturbations to fetal circulation compromise fetal growth and trigger the abnormal cardiovascular remodeling that underlies congenital heart defects. Computational modeling can be used to elucidate complex blood flow patterns in the fetal circulatory system for normal versus abnormal development. We present an overview of fetal cardiovascular physiology and its evolution from being investigated with invasive experiments and primitive imaging techniques to advanced imaging (4D MRI and ultrasound) and computational modeling. We introduce the theoretical backgrounds of both lumped-parameter networks and three-dimensional computational fluid dynamic simulations of the cardiovascular system. We subsequently summarize existing modeling studies of human fetal circulation along with their limitations and challenges. Finally, we highlight opportunities for improved fetal circulation models.

## 1. Introduction

**Figure 1:**
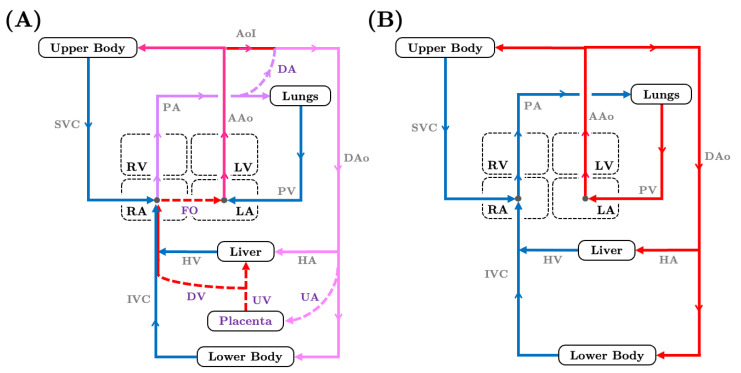
Blood flow in the fetal circulatory system (**A**) versus in the adult circulatory system (**B**). Dashed lines refer to shunts that are unique to fetal circulation. Arrows indicate flow direction. Line colors correspond to oxygenation levels with blue for oxygen-poor blood, red for oxygen-rich blood, and purple for mixed oxygenated and deoxygenated flow. Numerical values for oxygen saturation can be found in Appendix A. AAo—ascending aorta, DAo—descending aorta, PV—pulmonary vein, SVC—superior vena cava, PA—pulmonary artery, DA—ductus arteriosus, IVC—inferior vena vava, UA—umbilical arteries, DV—ductus venosus, UV—umbilical vein, RA/LA—right/left atrium, RV/LV—right/left ventricle, FO—foramen ovale.

The fetal circulatory network forms early to support the developing fetus and differs from the adult circulatory system notably in its oxygen and nutrient source as well as flow pathways that require a number of fetal shunts. Due to the nonfunctional nature of the fetal lungs, the placenta performs the necessary gas exchange, waste elimination and nutrient uptake. Three distinctive fetal vascular shunts—the foramen ovale, ductus venosus, and ductus arteriosus—emerge at approximately 4 weeks, 6 weeks, and 7–8 weeks gestation [1,2,3], firmly establishing the fetal vascular configuration that will persist up until birth (Figure 1A). Through the shunts, blood streams with different oxygen concentrations mix and are redistributed among vessel paths, affording the fetus flexibility and protection against adverse growth conditions. Following birth, pulmonary respiration engages as the fluid-filled lungs empty, the placenta detaches, and shunts rapidly regress as the cardiovascular system to adapts to its mature form (Figure 1B).

Fetal circulation is closely tied to fetal development [4]. Defects in the circulatory system disrupt gas and nutrient exchange as well as induce structural abnormalities to the heart and surrounding vessels as a result of altered cardiac loading. With an incidence of 3–7% [5], intrauterine growth restriction (IUGR) can happen when oxygen transport at the placenta is inadequate, leading the fetal circulatory system to operate in an altered manner that fails to deliver nutrients to different parts of the fetus [6,7,8,9]. Disturbances in flow patterns surrounding the developing heart may lead to a variety of congenital heart defects (CHDs) [4,10]. The prevalence of cardiovascular abnormalities pre- and post-birth together with uncertainties around the significance of maternal age and cardiac function on fetal development underscore the need for a deeper systematic understanding of fetal circulation and its connection to birth defects and pregnancy complications.

Much of our understanding of mammalian fetal circulation is derived from invasive injection experiments on large fetal animal studies performed in the early 20th century [11,12]. Advances in ultrasound and magnetic resonance imaging (MRI) have allowed for the routine clinical assessment of fetal hemodynamics and vessel morphology, yet these techniques either suffer from a lack of spatial–temporal resolution or predictive capability. When combined with information obtained from mathematical modeling, image-based computational simulations augment clinical measurements, shedding light on disease pathophysiology and helping to optimize clinical care [13,14,15,16,17,18]. Flow modeling allows for in silico studies of variations in parameters governing a system from vessel diameters to flow connections and implantation angles [19,20,21,22,23]. Due to their complex geometries and delicate nature, fetal circulation studies may especially benefit from the computational modeling of a predictive or exploratory in nature. Simplified computational models composed of idealized or reduced-order vessel models have already led to a better understanding of fetal diseases [24,25,26,27]. Progressive advancements in fetal MRI technologies continue to open up new possibilities for detailed patient-specific simulations of human fetal circulation. Three-dimensional (3D) patient-specific models of regions of interest within fetal circulation allow for accurate wall shear stress measurements, capturing a major driver of vascular growth and remodeling [4,21,28,29,30,31,32] and opening up the possibility of achieving a more in-depth understanding of normal and abnormal fetal development.

Small animal models such as guinea pigs and mice are practical alternatives to large animal models due to their rapid development, ease of manipulation and proximity to the human system [33,34,35], yet they are considered more basic science than clinical models due to the large differences in scale and physiology. Non-mammalian vertebrate embryos, namely chick and zebrafish, provide accessible models of heart and great vessel development, but they do not rely on continued maternal–fetal exchange [21,36,37,38].

This review summarizes current knowledge of human fetal hemodynamics and the role of imaging, large animal models, and computational models in shaping the field’s understanding of fetal circulation. Special attention is given to assessing computational models in terms of their degree of complexity and capabilities as well as highlighting future opportunities in the field. The body of the article is organized into four sections. Section 2, Fetal Cardiovascular Physiology, provides a background on fetal physiology and historical advances in measurement techniques to characterize cardiac hemodynamics. Section 3, A Primer on Computational Hemodynamic Simulations, acquaints readers with the theoretical foundations and assumptions of computational hemodynamic modeling. Section 4, Lumped Parameter Models of Fetal Circulation, reviews the application of reduced-order models of the fetal circulatory system to study fetal circulation in normal and complicated pregnancies. Lastly, Section 5, Local Hemodynamic Features of Fetal Circulation and 3D Simulations, draws attention to the relationship between blood flow patterns and important morphological regions within the heart.

## 2. Fetal Cardiovascular Physiology

**Figure 2:**
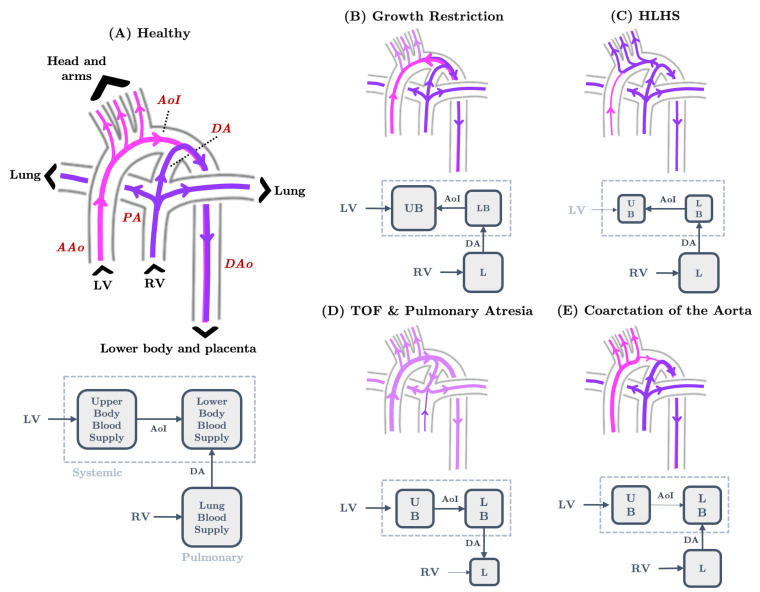
Blood flow path in the human fetal great vessel network as produced by 3D simulations. (**A**) Under healthy conditions, blood flow from the PA to the DAo through the DA and from the AAo to the DAo through the AoI. (**B**) In growth-restricted fetuses, AoI blood flow reverses due to increases in upper body blood supply caused by decreases in brain vascular resistance. (**C**) In HLHS fetuses, RV flow also supplies the upper body systemic circulation due to an underdeveloped LV, causing flow reversal in the AoI. (**D**) In TOF or pulmonary artresia, systemic circulation rescues the inadequate pulmonary blood supply, causing flow reversal in the DA. (**E**) With coarctation of the aorta, flow reveresal is not observed in the AoI or the DA; rather, AoI flow is greatly reduced due to aortic stenosis. Pathline color correlates with oxygen saturation with pink indicating a higher oxygenation level than purple. Note that left ventricular output is more oxygen-rich than right ventricular output (Appendix A). AAo—ascending aorta, AoI—aortic isthmus, DA—ductus arteriosus, DAo—descending aorta, HLHS—hypoplastic left heart syndrome, LV—left ventricle, PA—pulmonary arteries, RV—right ventricle, TOF—tetralogy of Fallot.

**Figure 3:**
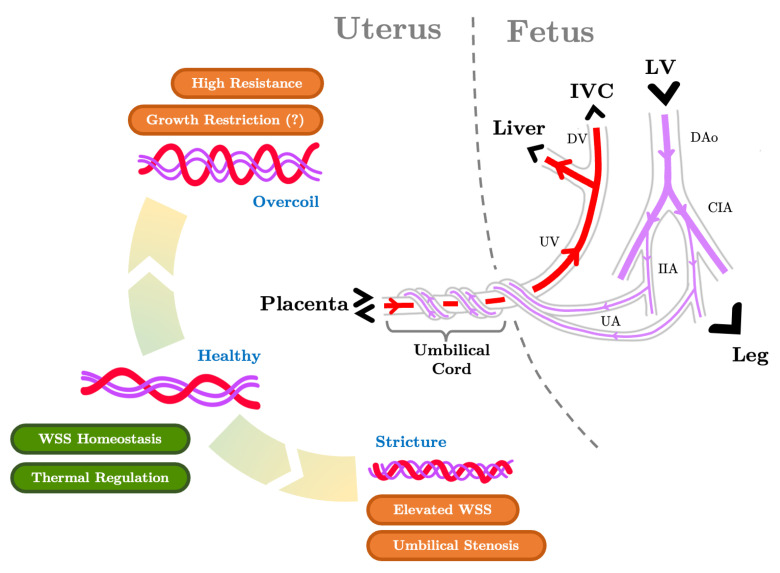
The anatomy and functional properties of the umbilical circulation network. The portion of umbilical arteries and veins outside of the fetal body is contained in the umbilical cord. The coiled geometry of the umbilical vessels maintains a stable mechanical stress environment as the cord contorts due to fetal motion and may contribute to fetal thermal regulation. When the cord is overcoiled (characterized by an elevated umbilical coiling index), vascular resistance increases, potentially implicating cord overcoiling in growth restriction. When the coil diameter reduces, umbilical cord stricture occurs and wall shear stress in the vessels increases, which may lead to stenosis and the formation of thrombosis. Line colors indicate highly oxygenated blood (red) and moderately oxygenated blood (pink) (Appendix A for values). LV—left ventricle, IVC—inferior vena cava, DV—ductus venosus, DAo—descending aorta, CIA—common iliac artery, IIA—internal iliac artery, UA—umbilical artery, WSS—wall shear stress.

Fetal circulation differs greatly from adult circulation in that there are not two distinct (oxygen-rich and oxygen-poor) flow pathways (Figure 1). Rather than gathering oxygen from the lung, oxygen-rich blood and nutrients are sourced from the placenta, entering the fetus through the umbilical vein that feeds directly into the liver [39]. A portion of the oxygen-rich blood is diverted through the ductus venosus to join with the oxygen-poor systemic venous return in the inferior vena cava, ultimately draining into the right atrium [40]. A stream of highly oxygenated blood flows into the left atrium through the foramen ovale, while a lower oxygen level stream enters the right ventricle [41]. Blood in the right ventricle is ejected into the main pulmonary artery, but the majority of flow (approximately 73% late gestation) passes through the ductus arteriosus into the descending aorta to supply the gut, kidneys, and lower extremities (Figure 2A) [42]. Oxygen-rich blood in the left ventricle is ejected into the ascending aorta and largely enters the vascular networks of the heart, brain, and upper limbs. Only a small portion (around 25% late gestation) of blood from the left heart crosses over the aortic isthmus, the segment of the aorta between the left subclavian artery and the ductus arteriosus, to join with right ventricular output in the descending aorta [42]. The umbilical arteries branch off of the iliac arteries (Figure 3) and channel blood into the placenta for nutrient exchange. Blood carrying carbon dioxide and other waste products drains into the inferior and superior vena cava.

The fetal heart is right-dominant with the right ventricle accounting for just below 60% of combined ventricular output in late gestation [42,43]. Due to the small volume of oxygen-poor flow returning from the lungs into the the left atrium, left ventricular flow is supplemented by the foramen ovale shunt, resulting in a slightly higher oxygen-rich left ventricular flow saturation, as compared to the right ventricle. Left ventricular output is largely retained in the coronary and cerebral (upper body) networks [12]. High fetal pulmonary resistance results in substantial right ventricular output flows through the ductus arteriosus to oxygenate the lower body and support placental circulation. Left ventricular output preferentially supplies the upper body (Figure 2A) [44]. Roughly one-third of the right ventricular output enters the pulmonary artery, while the majority of the right ventricular output is directed to the lower body and the placenta in a process unique to the fetal respiration. More metabolic waste accumulates in the right heart compared to the left heart, which works to adequately oxygenate lower body tissues and efficiently exchange nutrients with the placenta.

The human umbilical cord houses three vessels, two umbilical arteries and an umbilical vein (Figure 3). These vessels serve the critical role of maintaining fetal–placental circulation for gas exchange, metabolic waste clearance, and nutrient uptake. Umbilical arteries join the internal iliac artery on both sides of the placenta. The umbilcal vein connects the placenta with the liver. The three vessels meet at the umbilicus and extend out of the fetal body into the umbilical cord where they adopt a distinctive coil-like morphology. Postnatal analyses found that the average coiling index (number of umbilical artery coils per centimeter of cord length) is 0.21 in healthy pregnancies [45]. The coiling index is found through Doppler ultrasound analysis to be strongly correlated with umbilical flow indexes in midterm fetuses including resistance and peak velocity in midterm fetuses [46]. Deviations from the expected coiling index were associated with adverse perinatal outcomes [47], although clinical literature offers conflicting evidence as to whether low fetal body weight is associated with undercoiled or overcoiled umbilical vessels [47,48,49]. Umbilical cord stricture occurs when the diameter of the vessel coil becomes significantly reduced, often in conjuction with overcoiling; it is associated with umbilical vessel stenosis and can be a cause of fetal demise [50]. These findings indicate a clear connection between the structure and function of the umbilical arteries. Umbilical circulation also plays a pivotal role in maternal–fetal heat exchange, as 84.5% of excess heat generated can be eliminated through blood flow in the umbilical cord [51].

The morphological and hemodynamic properties of fetal circulation help maintain differentially oxygenated blood streams [44,52], ensuring an adequate supply of oxygen-rich blood to necessary organs [53]. Fetal shunts contribute to circulation adaptability, allowing for a high degree of flexibility in flow distribution and helping to maintain fetal vessel homeostasis or a state of equilibrium [11,12,44]. In addition to fetal circulation connectivity, flow to the brain, myocardium, lung, placenta, and other organs are modulated through vasodilation and vasoconstriction induced by neurological actions and vasoactive agents [54,55]. Abnormal flow reversal in the aortic isthmus may appear in fetuses with severe congenital heart defects (CHDs) of the left outflow tract including hypoplastic left heart syndrome, coarctation of aorta, and aortic stenosis (Figure 2) [27]. CHDs that impair the left heart function siphon flow from the right heart through the ductus arteriosus to the aortic arch in an attempt to rescue compromised cerebral flow. Similarly, when nutrient and oxygen supply to the fetuses is limited by placental insufficiency, the fetus preferentially increases flow distribution to the brain and heart by increasing peripheral vascular resistance and decreasing cerebral vascular resistance [56]. Changes to subsections of the vascular network have compounding effects on the rest of circulation, potentially leading to reverse and increased loading [57]. In the immediate term, vascular adaptation protects vital organs from hypoxic injuries. However, when prolonged, changes in vascular resistance may lead to an adverse remodeling of brain vasculature [56] or inadequate lower body development [58] due to lack of adequate blood supply.

### 2.1. Landmark Experimental Investigations of Fetal Circulation

Fetal animal models have played a pivotal role in advancing the clinical understanding of fetal circulation. Early experimental studies were frequently performed on sheep or pig fetuses due to their clinical translatability and easy access to the vessels of interest [33,59,60]. Sheep fetuses offer the additional advantage that their placentas are slow to separate, allowing for maintained fetal circulation after the fetuses are removed from the uteri [60]. As the placental structure of the sheep can be significantly different from that of the human, fetal lambs are generally less applicable to placental hemodynamic studies [33,39].

Prior to the early 20th century, the exact course of blood flow through the fetal circulatory system had not been definitively recorded. Several conflicting theories existed regarding the direction of flow through the foramen ovale and the degree of mixing of the two caval blood streams in the left atrium [59]. Early attempts to investigate foramen ovale flow began with injection experiments performed on live fetal animals extracted from the womb [11,12]. Through an injection study on pig fetuses, where a starchy suspension was injectected into live exposed pig fetuses, Pohlman (1909) proved that blood passes through the foramen ovale from the right to the left atrium [59]. Barclay, Barcroft, and colleagues (1939) injected radio-opaque media in fetal sheep and qualitatively described the distribution of fetal vena cava blood through the fetus [61]. Quantitative fetal cardiac flow split measurements became possible with the development of radioactively-tagged saline [34]. Intrauterine catheters allowed quantitative flow split measurements to be taken via radioactive microspheres injection while the fetus remained in utero [60].

### 2.2. Characterizing Fetal Hemodynamics through Ultrasound and Magnetic Resonance Imaging

While in vivo experiments provided a foundation for fetal circulation studies, such studies are limited to animal models due to their highly invasive nature. Detailed information regarding human fetal circulation was made possible by the development of fetal ultrasound [62,63,64,65,66,67,68,69]. FitzGerald and Drumm (1977) are credited with creating a reliable non-invasive technique to investigate fetal blood flow using a combination of echo and Doppler ultrasound [70]. Color flow imaging has also helped detail the hemodynamics of the fetal aorta, carotid artery, and smaller vessels [71]. Ultrasound measurements enable quantitative descriptions of human fetal cardiac output changes between week 12 and 34 of gestation [72] and organ-specific flow distribution changes between 10 and 40 weeks of gestation (Table 1) [73]. Doppler ultrasound assessments of blood flow velocities in various fetal vessels have further informed the pathophysiologies of numerous fetal conditions including intrauterine growth restriction [7,71,74], proteinuric pregnancy-induced hypertension [75,76], and twin–twin perfusion syndrome [77].

Despite their low cost and effectiveness, ultrasound-based methods are limited by poor spatial resolution, user-dependent errors, and complications related to material obesity [79,80,81]. Magnetic resonance imaging (MRI) offers a reliable, high-resolution, and versatile alternative. Applying MRI techniques to the fetus is a non-trivial task due to the fetus’s small size and unpredictable movements; multi-plane imaging and motion correction are required for high-fidelity volumetric reconstructions of the fetal heart and vessels [82,83]. MRI-based blood flow measurements in the fetus present additional challenges due to a lack of real-time ECG recording, which are required to correlate MRI data with heart and vessel motion in a cardiac cycle [84,85]. Metric optimized gating methods have been developed, enabling phase-contrast MRI (PC-MRI) measurements of blood flow rate across select vessel cross-sections in the human fetus (Table 2) [86] and non-invasive in utero assessments of 3D flow patterns in the human fetal heart and vessels [87,88]. By leveraging the different magnetic properties of oxygenated and deoxygenated hemoglobin, MRI could also provide non-invasive blood oxygen saturation measurement for fetal circulation (Table 2) [42,89].

## 3. A Primer on Computational Hemodynamic Simulations

### 3.1. Fluid Mechanics Principles of Blood Flow

Blood can be treated as an incompressible fluid whose motion is governed by the Navier–Stokes equations. Derived from the conservation of mass and momentum, the Navier–Stokes equations relate the 3-dimensional blood flow velocity field *v* and the scalar blood pressure *p* as functions of time *t* and space x,y,z [90]. The equations can be written as

(1) $$\begin{array}{cc} \nabla \cdot v & =0,\\ \rho \frac{\partial v}{\partial t}+\rho v\cdot \nabla v & =-\nabla p+\nabla \cdot \left(\mu \left(\nabla v+\nabla v^{⊤}\right)\right)+\rho f. \end{array}$$

ρ is the constant density of blood, f represents body forces, and μ is the dynamic viscosity of blood. Blood can be accurately approximated as a Newtonian (constant viscosity) fluid in larger vessels where blood experiences a high shear rate [91], in which case μ is constant, and $\nabla \cdot \mu \left(\nabla v+\nabla v^{⊤}\right)=\mu \nabla^{2}v$. For non-Newtonian fluids, where fluid viscosity changes with velocity, constitutive relations, such as the Carreau–Yasuda model, are needed to describe the dependence of viscosity μ on shear rate [36,92,93].

Cardiovascular computational simulations require a number of assumptions. Many hemodynamic simulations assume the vessel walls to be rigid and stationary throughout the cardiac cycle, negating any interaction that fluid may have on vessel wall motion and vise versa [94,95]. Fluid–structure interaction (FSI) models more accurately capture the effect of wall motion through mathematical coupling of the fluid and solid interfaces [96,97]. Popular FSI approaches include the Arbitrary Lagrangian–Eulerian formulation in which the fluid and solid domains deform together and are solved in their own domains [98] and the more simplified, computationally efficient, Coupled Momentum Method in which the solid domain is approximated as a membrane [99].

**Figure 4:**
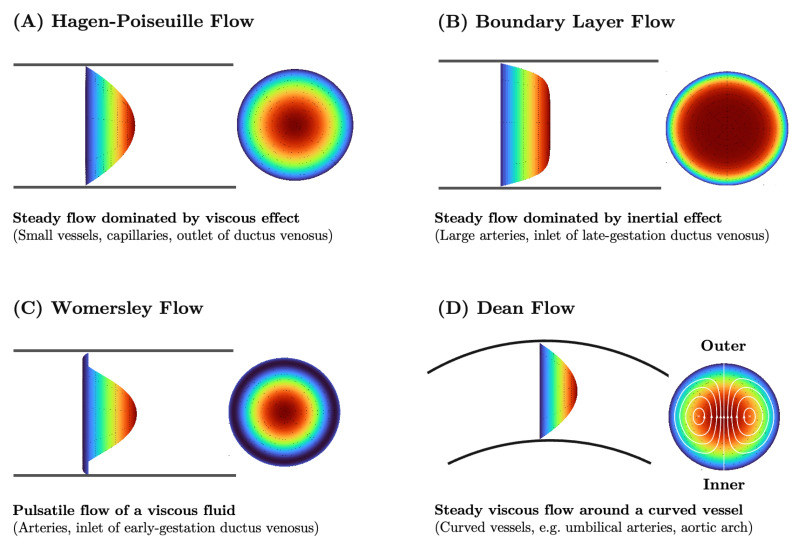
Hemodynamic flow profiles experienced in various cardiovascular simulations (**A**) Poiseuille flow is dominated by viscous effects and marked by a parabolic profile. Commonly seen in small vessels, capillaries, outlet of ductus venosus. (**B**) Boundary layer flow is dominated by inertial effect and marked by a rectangular, plug-like, profile. Often appears in large arteries, inlet of late-gestation ductus venosus. (**C**) Womersley flow exhibits a small amount of flow reversal near the vessel wall due to competing viscous and inertial forces. Typical of arteries and early gestation ductus venosus inlet flow. (**D**) Dean flow in minimally curved vessels produces a parabolic primary flow pattern as viscosity is the dominant phenomenon. A pair of counter-rotating vortices constitutes the secondary flow due to the combined effect of inertial and centripetal forces. Plots created via [101].

Blood flow itself takes on a different flow profiles as it travels through vessels of varying diameter and curvature (Figure 4). The Reynolds number (Re), a non-dimensional parameter comparing the inertial effects with that of viscosity, further characterizes expected flow behavior and is used to calculate both the Womersely number (pulsatile nature of flow) and Dean number (curvature effects). Steady laminar flow in a uniform, straight cylindrical pipe driven by a constant pressure drop follows the Poiseuille flow profile (Figure 4A), with a parabolic velocity profile elongated along the pipe centerline [90]. Poiseuille flow typically describes flow in small vessels. When the vessel curves, the bulk of viscosity-dominated flow is more likely to conform to the curvature of the vessel with minimal perturbations to its flow profile (Figure 4D). A Reynolds number much larger than 1 indicates that the flow is dominated by inertia and can become turbulent. Inertia-dominated flow in straight cylindrical pipes forms a boundary layer (Figure 4B), maintaining its principle flow direction and separating away from the vessel wall as it curves. The Reynolds number of blood flow in key locations in the fetal cardiovascular network is reported in Appendix B (Table A2). Womersley flow results from pulsatile flow in elastic vessels, in which curvature and cross-sectional shape non-uniformity have a negligible effect [100]. Standardized flow models such as Poiseuille and Womersley flow are used to predict how bulk hemodynamic properties are linked to vessel geometry and modulated by biological processes.

### 3.2. Three-Dimensional Hemodynamics Simulation

**Figure 5:**
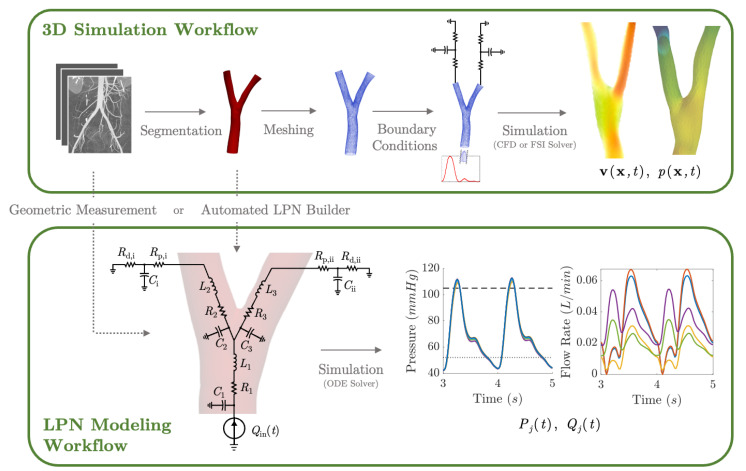
Computational workflow for patient-specific 3D or lumped parameter network (LPN) hemodynamic simulation. For 3D simulations, the steps include segmentation, meshing, defining of boundary conditions, and simulation. A time-varying flow curve is imposed at the inlet and a RCR Windkessel model representing downstream vasculature at the outlet. For LPN simulations, the Windkessel model can be constructed from geometric measurements or automatically from 3D vessel models. Large vessel segments are represented by RLC circuits, a small peripheral vascular network is represented by RCR circuits, and an inflow curve is represented as a current source.

The complex morphology of biological vessels can lead to a number of local hemodynamic flow features at junctions along the vessel walls or along the vessel centerline [102]. Local flow dynamics influence the mechanical stress environment, resulting in altered cardiovascular function, development, or disease propagation. Uncovering local flow features often requires solving Equation (1) or a related FSI problem numerically in an in silico 3D anatomical model of the area of interest (Figure 5). Idealized geometric models, in which simple shapes (cylindrical tubes, circular elbow bends, Y-joints, etc.) are used to reproduce key vessel characteristics, are often used for a generalized understanding of flow features. Patient-specific models recreate the anatomical characteristics that are unique to each individual. Three-dimensional (3D) anatomical reconstructions are typically constructed by segmenting volumetric images obtained from high-resolution imaging, such as computed tomography or MRI (Figure 5).

Through meshing, the 3D anatomical model is broken down into a grid or collection of smaller blocks (Figure 5) on which the governing equations can be solved in a more simplified manner [103]. Before conducting numerical simulations and solving the governing equations, boundary conditions (BC) must be defined [99,104]. Inlet BCs prescribe a time-varying inflow velocity profile, which is typically directly measured using Doppler ultrasound or PC-MRI [21,38,105,106]. Outlet BCs, which are typically pressure boundary conditions, represent the hemodynamic effects of downstream vasculature. All outlet BCs can take the form of lump parameter circuits [106,107,108]. Parameters used to define the BCs must be carefully tuned to match clinical or experimental measurements such as cardiac output, blood pressure or flow split [21,38,109,110]. Following validation, critical hemodynamic properties that are difficult to obtain clinically/experimentally such as wall shear stress (WSS) and oscillatory shear index can be calculated and analyzed.

### 3.3. Lumped Parameter Networks

The cardiovascular system is a closed network of interconnected vessels. Depending on the question at hand, computationally expensive 3D simulations are not always warranted. Lumped parameter networks (LPNs) or reduced-order models that capture the global connection and behavior of a desired vascular network can provide useful insight into general pressure and flow trends. LPNs abstract away the spatial variability in flow and pressure profiles within individual vessels, rendering them functions of time alone [104]. Zero-dimensional lumped parameter models equate blood flow through a vascular network with that of current running through an electric circuit. Each circuit component captures a phenomenon relevant to cardiovascular flow. Flow dissipation is represented by a resistor, vessel wall compliance is represented by a capacitor, and the inertia of blood flow is represented by an inductor (Table 3). After integrating Equation (1) over its three spatial dimensions, a generic vessel segment can be modeled as an RLC circuit (Figure 5) given the following assumptions: (1) blood is Newtonian, (2) blood flow is parallel and axisymmetric, following the Poiseuille velocity profile, (3) the convective acceleration of blood flow is negligible, (4) the vessel wall is linearly elastic, (5) elastic waves propagate rapidly in the blood vessel [111]. This analysis relates the upstream and downstream pressure ($P_{i}\left(t\right)$, $P_{i+1}\left(t\right)$) and flow rate ($Q_{i}\left(t\right)$, $Q_{i+1}\left(t\right)$) in a vessel segment as

(2) $$\begin{array}{cc} Q_{i}-Q_{i+1} & =C\frac{dP_{i}}{dt}, \end{array}$$

(3) $$\begin{array}{cc} P_{i}-P_{i+1} & =L\frac{dQ_{i+1}}{dt}+RQ_{i+1}. \end{array}$$

In particular, the capacitance (*C*), resistance (*R*), and inductance (*L*) are given by

(4) $$C=\frac{2\pi r^{3}\left(1-\sigma^{2}\right)}{Eh}\approx \frac{3\pi r^{3}}{2Eh},\;R=\frac{8\mu l}{\pi r^{4}},\;L=\frac{\rho l}{\pi r^{2}}.$$

Here, σ≈0.5, *E*, and *h* are the Poisson ratio, Young’s modulus, and thickness of the vessel wall [112]. ρ and μ are the density and viscosity of blood. *l* and *r* are the length and cross-sectional radius of the vessel segment, which can be taken from reported measurements or extracted from 3D models (Figure 5).

The RLC vessel “building block” can be easily adjusted to represent varying vessel properties [111,113]. Rigid small vessels will see the capacitance and inductance set to zero so that the vessel is represented by a resistor alone. A nonlinear resistor is used to model irregular hemodynamics associated with sudden changes in vessel diameter, separated flow, and heart valves [114,115,116]. When using a nonlinear resistor, an additional term $KQ_{i+1}^{2}$ is added to the right-hand side of Equation (3), where $K=\rho k/2\pi^{2}r^{4}$ and *k* is an empirically determined constant following the classic hydraulics principle [116]. Parameters are generally tuned to match clinical observation [24,117].

LPN models can be open-loop or closed-loop depending on if the entire (closed-loop) or only a portion (open-loop) of the circulatory system is represented. Open-loop LPNs have at least one inlet and one outlet. Inlets are typically connected to current sources capturing the measured inlet flow rate, and the outlets are grounded to RC or RCR circuits describing peripheral organ vascular beds (Figure 5). Closed-loop models represent the circular nature of the cardiovascular system and typically include both arterial and venous segments. Peripheral vascular beds are modeled by an RCR circuit with one end connected to an arterial element and the other connected to a venous element. Closed-loop models typically account for cardiac activity with equivalent circuits representing heart chambers that can be tuned to generate expected cardiac outflow measurement, making use of a time-varying elastance element that represents the changes in myocardium stiffness in a cardiac cycle (Table 3) [118,119]. Valves are typically modeled as nonlinear resistors connected in series to diodes that enforce unidirectional flow (Table 3) [113].

LPNs produce a system of ordinary differential equations of pressure and flow rate over time *t* at every vessel bifurcation point, and they can be solved using a variety of time-marching numerical methods such as explicit or implicit Euler methods and Runge–Kutta methods (Figure 5) [120]. The results simultaneously describe how flow rate and pressure at junctions between vessels and organ-specific vascular beds change over a cardiac cycle.

## 4. Lumped Parameter Models of Fetal Circulation

Lumped parameter models offer a non-invasive method of capturing organ-specific and global attributes of fetal circulations, allowing for the study of how specific anomalies affect the whole fetal circulatory network. Such simulations can inform fetal circulatory physiology and pathophysiology. LPNs are computationally inexpensive to solve, with simulations being solved within a matter of minutes on a standard computer. The omission of spatial heterogeneity in blood flow masks local flow characteristics.

### 4.1. Evolution and Sophistication of Fetal Lumped Parameter Network Models

**Figure 6:**
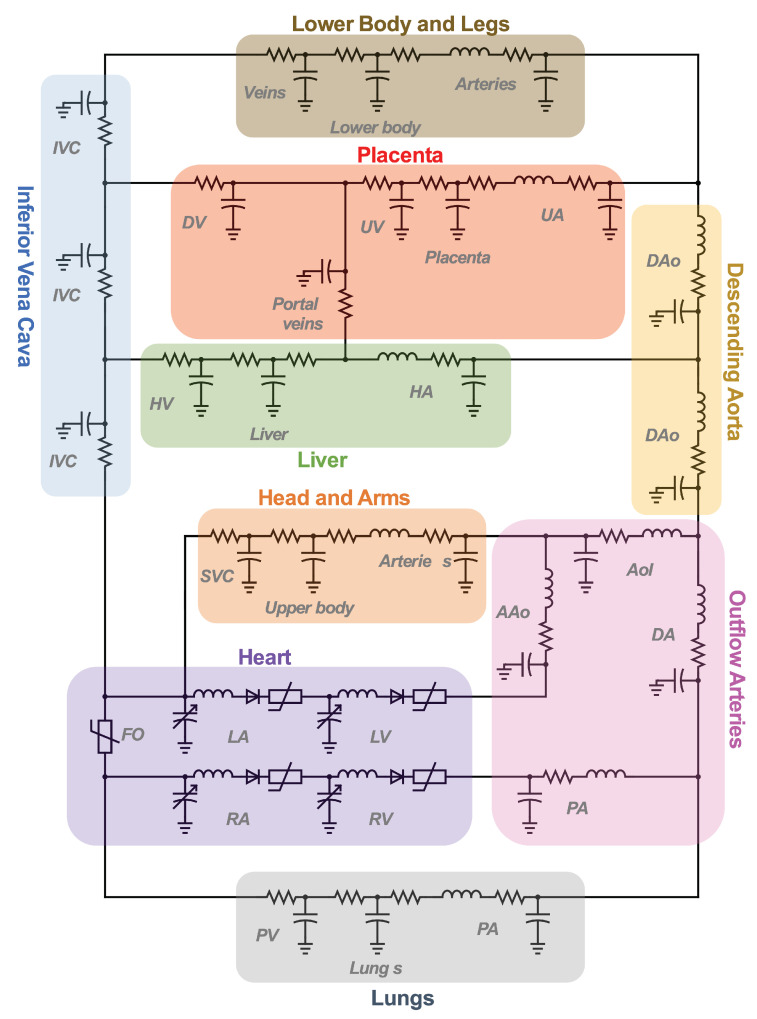
Representative fetal LPN circuit. Note how it relies heavily on RLC components as a basic vessel building block.

The first LPN model of human fetal circulation was constructed to describe the cardiovascular system of a 38-week fetus with a body weight of 3 kg [121]. The LPN consisted of a fetal heart model connected to a network of 19 vessel segments and capillary bed (Figure 6). Parameters defining the LPN were either adopted from reported values based of the fetal lamb with the same body weight or calculated from existing ultrasound measurements of human fetal vessel geometry. The accuracy of the results was assessed by comparing the simulated flow rate with those obtained using Doppler ultrasound with a difference of less than 20% considered accurate. The model provided a comprehensive estimate of human fetal blood pressure throughout the circulatory network, which was an insight previously inaccessible due to the invasive nature of direct fetal pressure measurement techniques [122,123,124].

To improve the utility of LPN fetal hemodynamic models, changes in body weight due to gestational stage and natural variability among fetuses must be considered. The allometric scaling principle stipulates that vessel dimensions (*Y*) scale with body weight (*W*) raised to an empirically determined power (α), that is

(5) $$Y\propto W^{\alpha }.$$

Capper et al. used cube-root (α=1/3) scaling laws to construct an LPN model of the human fetal systemic arteries and examine how umbilical flow changes between week 28 and week 40 of gestation [125]. Simulation results revealed linear decreases in the umbilical artery pulsatility index and resistance index with increasing gestational age. Both indexes were higher on the fetal end than on the placental end, but the differences progressively decreased as the fetus aged. Cubic-root allometric scaling laws assume uniform growth (proportional to body surface area) and do not account for the unique remodeling courses in all fetal vessels. Empirically derived allometric scaling laws provide a more comprehensive assessment of fetal hemodynamics changes throughout the circulatory system from week 20 to week 38 [114]. Notably, LPN simulations using empirically derived allometric scaling laws show increased pulsatility in the throacic aorta and decreased pulsatility in peripheral arteries. Peak flow velocities through the ductus arteriosus almost double from 20 weeks to 38 weeks, while those in the ductus venosus only see minimal increases. Empirically derived LPN results match experimental observations [126,127,128]. Including a model for hepatic vascular resistance, based on the fractal-like structure of the liver’s capillary network and scalable with hepatic volume, can provide a more sophisticated description of the changing fetal hemodynamics during gestation [129]. A detailed umbilical–hepatic circulation LPN, coupled to a resistor network model of the surrounding veins, revealed that the degree of ductus venosus shunting (proportion of umbilical vein flow entering the ductus venosus) nearly halved across the latter half of gestation, drastically declining from week 20 to 28. The model highlights that the degree of ductus venosus shunting remains relatively unaltered with changing hemocrit, pressure drop, and umbilical cord length, suggesting that changes in ductus venosus shunting during normal gestation are likely the result of anatomical remodeling of the vessel. Insights gained from these LPNs continually increase the field’s understanding of fetal circulation.

### 4.2. LPN Hemodynamic Models of Growth-Restricted Fetuses

LPN hemodynamic models allow for the perturbation of a specific hemodynamic property in isolation and delineate its effect on the cardiovascular system. Intrauterine growth restriction (IUGR) is a fetal cardiovascular disease stemming from a number of pathologies (elevated placental resistance, decreased brain vascular resistance, dilated ductus venosus, etc). Insights into the isolated impact of each factor on fetal circulation are made possible through LPNs.

In an LPN of fetal arteries and peripheral vessels of a 33.2-week healthy human fetus, a four-fold increase in placental resistance and four-fold decrease in brain resistance were implemented to recapitulate aspects of IUGR pathology [24]. Modulations in placental and brain resistance induced end-diastolic reverse flow in the aortic isthmus, significantly diverting lower-body blood flow to the upper-body for a “brain-sparing effect”. Brain resistance decreases were much more strongly associated with enhanced cerebral blood flow than placental resistance increases, suggesting that brain sparing is likely the result of a compensatory mechanism independent of placental vascular pathology. A similar study showed that the pulsatility index in the umbilical arteries and cerebral vessels is sensitive to brain and placental resistance and that umbilical artery flow reversal emerged with a three-fold increase in placental resistance [130]. To study the hemodynamic implications of ductus venosus dilation in IUGR, Pennati et al. used a LPN model of a 38-week-old human fetus’ circulatory system ([121]) and modified ductus venosus parameters to create a 30–150% dilation [131]. Progressive ductus venosus dilatation was associated with the progressive suppression of end diastolic flow and enhanced time-averaged flow in the ductus venosus. Umbilical vein flow was augmented to a lesser extent, thereby increasing the amount shunted into the vena cava. Pennati et al.’s simulation results mimicked hemodynamic changes in growth-restricted fetuses observed clinically using Doppler ultrasound. These results suggest that the fetus may induce dilation in the ductus venosus to ensure adequate oxygen delivery to the fetus in hypoxic conditions.

### 4.3. Patient-Specific Parameter Estimation in LPN Models for Diagnosis

LPNs can be used to estimate clinically relevant hemodynamic parameter values in individual patients. Parameters such as peripheral vascular resistance and pressure in specific vessels can be highly informative and cannot be directly measured through non-invasive means (Table 1 and Table 4). Using LPNs, parameters are iteratively tuned to accurately reproduce clinically measured values [18,132]. The process of tuning lumped parameter values to match clinical measurements may also be referred to as an “inverse solution” of a LPN. The resulting LPN model constitutes a patient-specific representation of an individual’s cardiovascular system.

Hemodynamic indices estimated using LPNs offer quantitative insights on fetal vascular physiology and pathology. The non-invasive measurement of aortic pressure in individual fetuses can be accomplished by tuning an LPN model of the fetal lower-body vascular system to match ultrasound aortic flow and pulsation measurements [133]. Struijk et al. used this technique to estimate pressure values in 21 healthy fetuses from 20 to 40 weeks gestation, obtaining a linear increase in systolic aortic pressure from 37 mmHg at 20 weeks to 58 mmHg at 40 weeks and a linear increase of mean pressure from 28 to 45 mmHg. Peripheral vessels experienced a four-fold exponential decrease in resistance and an eight-fold exponential increase in compliance in the last 20 weeks of gestation. Through an analogous technique, IUGR fetuses were found to have a significantly increased placental vascular resistance and compliance and significantly decreased coronary artery resistance, cardiac output, placental flow, and brain resistance [134,135]. Changes in placental resistance were noticeably more substantial than those of brain resistance [24] when comparing vascular adaption in healthy and IUGR fetuses. These findings highlighted an essential mechanism of fetal circulatory adaption in IUGR patients. Similarly, the ultrasound-derived LPNs of fetuses whose mothers had diabetes mellitus (FMDMs) showed that placental resistance and cerebral artery diameters significantly decreased, while cerebral vascular resistance and aortic diameter significantly increased [136] when compared to healthy fetuses, with differences exacerbated by increased gestational stage. The morphological and hemodynamic changes uncovered through the LPN correlate with enhanced placental flow and reduced cerebral flow, suggesting a mechanism for why FMDMs tend to have an enlarged placenta and underdeveloped brain.

Hemodynamic measurements obtained from tuned LPNs help inform fetal health assessments and patient stratification. LPN-based non-invasive fetal aortic pressure measurements can systematically be adopted to assess the fetal well-being of mothers with pregnancy-induced hypertension and pre-eclampsia [133]. Ultrasound-derived LPNs of IUGR fetuses revealed that severe cases of IUGR exhibit a higher degree of brain resistance decrease and a drastically increased placental resistance [24]. These specific vascular resistance values can be used as more reliable predictors of adverse perinatal outcomes associated with IUGR. Doppler-derived flow indexes alone predicted adverse perinatal outcomes with a 73% sensitivity, and adding model-derived placental resistance, coronary resistance, cerebral resistance, and placental compliance improves the sensitivity to 91% [134,135]. The parameter tuning of fetal LPNs lays the foundation for the in silico planning of fetal surgeries, helping clinicians predict an individual’s response to particular treatment courses [18,132].

### 4.4. LPN Models of Transitional Hemodynamics in Neonates

LPN fetal circulation models can be adapted to investigate transitional hemodynamics in neonates. Transition from fetal to adult circulation is characterized by the gradual closing of the shunts, detachment of the placenta, and the significant enhancement of pulmonary blood flow as the lungs expand [137]. Characteristic fetal vascular structures persist for minutes to hours after birth. The evolution of fetal vascular structures is captured via time-dependent functions tuned to match experimental observations [138]. Fetal to neonate vessel evolution has previously been incorporated into a hemodynamics-respiratory LPN model through the addition of a mass-balance model of oxygen concentration dynamics that accounts for oxygen transport driven by blood flow, uptake at the lung and placenta, and consumption by the organs [78]. The hemodynamics–respiratory LPN model was used to evaluate the effect of umbilical cord clamp timing on neonatal cardiovascular and respiratory performance [78,139]. The model was validated against experimental flow measurements obtained from key vessels [43,72,140]. Compared to immediate cord clamping (ICC), simulated delay cord clamping (DCC) in a healthy full-term fetus produced an 11.7% increase in neonatal blood volume, 20% increase in cardiac output, and 27% faster attainment of adequate peripheral oxygen saturation [78]. The potential benefit of DCC was further tested on LPNs of infants born preterm [139] as well as with conditions such as patent ductus arteriosus, respiratory distress syndrome, and growth restriction. If delivered with ICC, 20–33 week premature infants without further complication were predicted to experience a 15% loss of blood volume, and those delivered between 32 and 40 weeks may experience a 10% loss. The 30-week preterm neonates delivered with ICC were predicted to have a cerebral oxygen saturation dropping below the fetal level for one minute after birth, while those delivered with DCC did not experience cerebral hypoxia. DCC was determined to be especially beneficial for otherwise healthy premature infants with a lower gestational age at delivery as well as 30-week preterm infants with growth restriction, patent ductus arteriosus, or respiratory distress. Simulations revealed that DCC slowed down the degradation of placental flow, allowing for the redistribution of placental flow and prolonging of oxygen uptake at the placenta [78]. The DCC model also saw higher and more persistent ductus arteriosus flow shunting, which is associated with a more efficient transition to pulmonary circulation.

## 5. Local Hemodynamic Features of Fetal Circulation and 3D Simulations

The 3D nature of the vascular system plays an important role in local hemodynamic behavior. Capturing local hemodynamic stresses, flow regimes and in some instances severe preessure drop requires a spatiotemporal resolution not present in LPN simulations. There are a number of points within the fetal circulatory system where multiple bloodstreams meet or diverge, producing complex local flow features that cannot be captured in reduced-order models. The controlled interactions of these bloodstreams ensure the appropriate distribution of oxygen and maintain a suitable mechanical stress environment for cardiovascular development. Detailed spatiotemporal flow information at various vessels of interest is particularly relevant for fetal circulation research, necessitating the use of 3D computational fluid dynamics simulations. When coupled to closed-loop lump-parameter bounds, multiscale simulations can reveal how changes within a 3D region of interest affect the rest of circulation [18].

### 5.1. Blood Flow in Healthy Fetal Hearts and Fetal Hearts with CHDs

Targeted computational investigations into fetal cardiac hemodynamics can lead to a better understanding of its unique form and function, including fast heart rate, small size, and higher load in the right heart. Intracardiac flow takes on complex spatiotemporal patterns throughout development [141,142]. At 20 weeks gestation, blood enters the ventricles at a high velocity during diastole, forming a region of low pressure around the aortic and pulmonary valves. Two flow spirals (vortex rings) successively emerge near the atrioventricular valves during early diastole (E wave) and peak diastole (A wave). During systole, blood rotating around the vortex ring is ejected into the outflow arteries to produce a high-velocity helical flow profile. Numerical results were in line with Doppler ultrasound measurements [62,126,143]. The kinetic energy stored in the vortex structures is hypothesized to aid the pumping of blood during systole, but energy analyses of fetal right ventricle blood flow do not definitively support this [141]. The significance of these high-momentum vortex rings likely lies in their ability to impose increased wall shear stress to the ventricular surface along its path, maintaining a stable mechanical environment that may be critical in cardiac growth and remodeling [144]. Patient-specific simulations can help validate these hypotheses.

The human fetal heart can be reliably imaged using ultrasound beginning at mid-gestation [145]. Despite the rapid fetal heartbeat, rigid wall simulations of the heart wall (myocardium) can lead to a number of insights [146,147]. Depending on the question being asked, FSI simulations may prove more appropriate. Four-dimensional (4D) ultrasound image sequences allow for patient-specific anatomical reconstructions as well as ventricular wall motion encoding [142,148]. The 4D-ultrasound-based simulations also allow ventricular motion patterns to be modified in silico to study the effect of contractile behavior changes on fetal intraventricular hemodynamics. Peristaltic motion from the tricuspid valve inlet to the pulmonary outlet begins to emerge in some healthy 20-week human fetal hearts. Numerical simulations have augmented this knowledge by capturing a decrease in pressure drop between the tricuspid valve inlet and pulmonary outlet as well as reduced systolic right ventricular work output [149]. Simulations also showed that ventricular torsion had minimal impact on intraventricular flow pattern or energy dynamics in healthy 22-week human fetal left ventricles [150].

**Figure 7:**
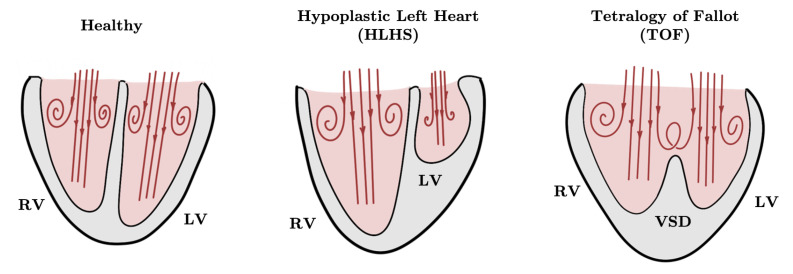
Schematic of diastolic blood flow and vortex patterns in fetal ventricles. In the healthy fetal heart, diastolic vortex rings are attached to the ventricular walls, generating high WSS. With hypoplastic left heart syndrome (HLHS), the diastolic vortex ring in the underdeveloped left ventricle does not contact the ventricular wall, so left ventricle WSS is persistently low. With tetralogy of Fallot (TOF), diastolic vortex rings in the two ventricles interact due to flow across the ventricular septal defect (VSD), applying high WSS on the ventricular septum. LV—left ventricle, RV—right ventricle.

In fetuses with hypoplastic left heart syndrome (HLHS) and tetralogy of Fallot (ToF), 4D-ultrasound-based patient-specific blood flow simulations revealed abnormal intracardiac flow patterns associated with each disease phenotype (Figure 7). HLHS hearts have grossly thickened left ventricular walls and underdeveloped left ventricles that are inadequate in supporting systemic circulation. Simulations of week 22 to 32 HLHS fetal heart hemodynamics showed that the diastolic inflow jet produces a single narrow vortex ring that propagates rapidly toward the left ventricular apex without interacting with the wall [25]. As a result, a large number of HLHS left ventricles show reduced wall shear stress throughout the cardiac cycle compared to controls. Cardiac outlet stenosis in HLHS hearts results in a largely stagnant left ventricular blood pool. The right ventricles of HLHS fetal hearts must, therefore, take on increased loads, resulting in increased right ventricular volume and output. Despite significant flow changes, the mechanical stress environment and energy dynamics remain at a homeostatic state with no significant difference seen in the remodeled right ventricle as compared to the control [151], implying that the heart underwent structural remodeling to maintain these values in equilibrium.

TOF is characterized by right ventricular hypertrophy, pulmonary outflow tract stenosis, enlarged aortic valve, and ventricular septal defect [26]. Hemodynamic simulations of week 22 to 31.5 ToF fetal ventricles reveal higher systolic pressure in both ventricles and increased wall shear stress in the right ventricles. Large ventricular septal defects allow flow shunting between the ventricles, disrupting the diastolic vortex rings and exposing the ventricular septum to elevated wall shear stress. Intraventricular vortex structures differ greatly among individuals as ToF phenotypes are highly variable. These differences underscore the necessity for simulation studies to account for patient-specific geometry.

### 5.2. The Great Vessels and Cardiac Output Distribution

Bloodstreams split and converge in the fetal outflow arterial network (Figure 1). Under physiological conditions, the ductus arteriosus shunts blood from the pulmonary arteries to the aorta (right to left). Flow in the aortic isthmus connects the aortic arch to the descending aorta. Flow conditions can be altered in abnormal (disease) conditions, in which case the ductus arteriosus will act as a fail-safe mechanism to mitigate the adverse effects (Figure 2).

Complex flow patterns due to interacting flow streams emerge in late-stage gestational models of the fetal aorta, pulmonary arteries, and ductus arteriosus. In the healthy state, flows in the fetal aortic arch and ductus arteriosus form a pair of counter-rotating Dean vortices resulting from vessel curvature (Figure 4) [152]. Ductus arteriosus blood flows at a high velocity onto the the distal aortic wall and curves downward into the descending aorta with no noticeable flow diversions [27]. A small portion of left ventricular blood enters the descending aorta through the aortic isthmus during systole, flowing perpendicularly to the ductal jet and streaming largely along the proximal wall of the descending aorta. Flows in the pulmonary arteries and descending aorta stream unidirectionally during the systole and swirl during the diastole. Flow dynamics were obtained numerically, and results were validated against in vitro experiments [27].

The in silico modulation of vessel morphology or hemodynamics can inform the pathology of various cardiovascular defects (Figure 2C–E). The 3D computational simulations of the ductus arteriosus connected to the aorta and pulmonary system have helped define ductus arteriosus functionality [27]. In an HLHS case study, the great vessels of healthy patients were modified in silico to incorporate HLHS morphological hallmarks. Altered great vessel morphology led to altered ductus arteriosus flow into the aorta (both the arch and descending aorta) and a two-fold increase in ductual flow rate. Recreating right ventricular output restriction associated with pulmonary atresia showed reverse flow in the ductus arteriosus with flow from the aortic arch draining into the pulmonary network. Similarly, in TOF, limited pulmonary flow was supplemented by blood from the aorta, causing systolic flow reversal in the ductus arteriosus. These observations suggest that the ductus arteriosus connects the pulmonary and systemic circulatory system, allowing compromised circulation on one side to be supplemented by the other. Similarly, computational hemodynamic simulations have been used to detail changes in aortic isthmus flow under pathological conditions.

In patients with coarctation of the aorta, where the aortic isthmus is stenotic, hemodynamic indices are insensitive to small in silico reductions in aortic isthmus diameter [152]. When the aortic isthmus diameter is reduced more than 55%, the velocity and wall shear stress increase exponentially, while pressure exponentially decreases. Flow characteristics in the aortic isthmus change noticeably with the disappearance of helical flow and absence of flow reversal. A reduction in aortic inflow rate greater than 60% leads to aortic isthmus flow reversal [153]. A backflow jet originating from the ductus arteriosus collides with the forward-flowing bloodstream in the aortic arch, forming a stagnation zone that moves increasing upstream with progressively reduced aortic inflow diameters. Ductus arteriosus and aortic isthmus wall shear stress increase with increasing degrees of backflow. The results of these in silico vessel reductions support the hypothesis that aortic isthmus flow reversal helps restore left and right ventricular output equilibrium. Increased wall shear in the ductus arteriosus is also hypothesized to trigger the migration of ductus arteriosus endothelium into the aorta, potentially contributing to the initiation and progression of coarctation of the aorta and interruption of the aortic arch. These in silico results could serve as a basis for experimental perturbations that take into account computed force values and spatially correlate them with cellular and molecular changes.

### 5.3. The Umbilical Arteries and the Role of their Helical Morphology

The human umbilical arteries possess a unique spiral shape. Theoretical investigations of viscous flow in curved and coiled pipes revealed the formation of counter-rotation bi-helical flow patterns driven by centripetal forces (the so-called “Dean flow”, as shown in Figure 4) and the dependence of flow properties on pipe curvature and torsion [154,155]. Recent computational simulations of blood flow in helical pipes resembling umbilical cord geometry have examined these distinctive flow features in the context of fetal physiology [156,157].

Several computational hemodynamic studies utilize idealized umbilical artery geometries where the artery is represented by a straight helical rigid tube with uniform circular cross-sections. By varying this idealized form, umbilical artery flow dependence on coiling parameters [156] and vessel dimension [157] can be elucidated. A bi-helical Dean flow profile was obtained for all coil and vessel diameter variations with more prominent vortexes obtained for arteries of lower pitch, i.e., less distance between each pair of coils. Artery models with more coils, smaller diameters, and longer cord length imposed a higher resistance to blood flow, implicating these umbilical artery features in growth restriction and other fetal diseases. The increase in vascular resistance was more sensitive to an increase in the number of coils than a decrease in their pitch [156]. Wall shear stress along the helical section increased noticeably with reduced pitch and coiling diameter, offering a possible mechanistic understanding of how umbilical cord stricture can lead to thrombosis and stenosis (Figure 3). The faster systolic flow seen in IUGR fetuses imposed higher peak wall shear stresses on the umbilical artery, although the averaged wall shear stress was not significantly different [158,159].

The umbilical artery anatomy varies considerably in both healthy and adverse conditions. Vascular resistance and wall shear stress stay relatively constant in uniform spiral vessels over a wide range of bending curvatures [160]. The helical umbilical arteries also maintain a steady pressure gradient [161]. The umbilical arteries may have adapted their helical shape to achieve stable hemodynamic stress levels given the constant contortion of the umbilical cord with fetal movement. In extreme cases, umbilical cord knotting or pathological remodeling may cause the umbilical arteries to become stenosed. Pulsatile blood flow simulations of locally constricted umbilical arteries show flow separation at the constriction along with downstream vortex formation [162]. Vortex eddies produce low-velocity regions around the umbilical artery centerline downstream of constriction. Three-dimensional (3D) simulations of blood flow in locally constricted helical umbilical arteries demonstrate that pressure upstream of the constriction increased exponentially with degrees of diameter reduction and decreased immediately downstream of the constriction [161]. A region of reverse flow is present downstream of the stenosis, but the helicity of the vessel confines the vortexes to the inner convex wall.

Computational simulations have also been used to non-invasively study the role of umbilical vessels in human fetal–maternal heat exchange [163]. Typically, heat transfer between blood vessels and their surrounding tissues is captured through a governing equation that takes into account heat conduction, convection through blood flow and metabolic heat generation, among other things. [164,165,166,167,168]. Fetal thermoregulation uses a simplified model with steady heat conduction between the umbilical vessel and the amniotic fluid. Results of fetal–maternal heat exchange simulations showed that the temperature of blood in the umbilical artery decreased roughly linearly from 37.5 °C at the fetal end to 37.235 °C at the placental end, while blood in the umbilical vein was around 37.2 °C throughout, suggesting that its helical structure also accelerates heat dissipation to facilitate thermoregulation in the fetus.

### 5.4. The Ductus Venosus and Placental Venous Return Distribution

Fetal veins carry oxygen-rich blood from the placenta (via the umbilical vein and ductus venosus) with oxygen-poor systemic venous return, often without physical barriers separating the two types of blood (Figure 1A). In spite of the lack of barrier, blood with disparate oxygen concentration levels do not fully mix, which is a key element to the proper distribution of materials in the fetus. The parallel flow streams of oxygen-rich ductus venosus blood and oxygen-poor vena cava blood have been captured via 4D-MRI with oxygen-rich blood flowing preferentially from the thoracic vena cava across the foramen ovale and eventually reaching the ascending aorta [52,87]. A disproportional abundance of highly oxygenated blood in foramen ovale flow produces an increased oxygen level in the left heart, prioritizing the heightened metabolic needs of the heart and brain.

The ductus venosus assumes a unique trumpet-like form that is narrower at the umbilical end and wider at the caval end. Its structure is subjected to rapid blood flow. Flow simulation of the umbilical vein, left portal vein, and ductus venosus in a healthy 34-week fetus captured a skewed inflow jet into the ductus venosus that induced vortex formation along the wall proximal to the left portal vein [169]. The vortex structures are attenuated downstream, and the flow profile assumed a skewed parabolic shape. A fluid–structure interaction study of a similar model for late-gestation fetal veins showed blunt flow profiles and boundary layer formation (Figure 4B) near the ductal inlet that gradually morphed into a skewed parabolic flow profile toward the outlet [170]. in silico wall motion at the ductus inlet matched pulsation patterns captured through ultrasound [171]. The transition from inertia- to viscosity-dominant flow behavior can be attributed to the reduction of flow velocity in the ductus venosus that is dependent on the vessel’s trumpet-like shape [172]. The 11 to 13-week fetuses have thinner veins that experience slower flow, rendering ductus venosus flow viscosity-dominant. Computational simulations support the parabolic and largely symmetric flow profiles about the central axis with Womersley-like flow emerging during atrial contraction with the large fluctuation in ductus venosus inflow velocity [173].

Hemodynamic simulations underscore the connection between the morphology of the ductus venosus and its functional properties. Pressure in the ductus venosus can be calculated from flow simulation as a means of estimating fetal central venous pressure. In late gestation, the pressure drop between the ductus venosus outlet and umbilical vein inlet is found to be sensitive to the branching angle values between the two vessels [169]. Since the ductus venosus is critical in diverting oxygen-rich blood to the heart, flow split at its junction with the umbilical vein is of considerable interest. Hemodynamic simulations show that the conicity of the ductus venosus and branching angle and umbilical vein are strong determinants of ductal flow behavior, and they must be considered to achieve reliable estimates of ductus venosus flow rate [174]. For a healthy late-gestation fetus, simulations indicated that approximately 54% of umbilical blood is shunted through the ductus venosus, as confirmed by Doppler studies [175]. Shunt strength is relatively robust against perturbations to vessel geometry and umbilical inflow behavior [170]. However, if the ductus venosus is a uniform cylindrical tube (without conicity), shunt strength decreases substantially to approximately 30%, suggesting that the trumpet-like shape of the vessel may have evolved to ensure abundant oxygen-rich blood supply to the fetal heart.

## 6. Concluding Remarks and Future Directions

We summarized key systemic properties and local blood flow features of the human fetal circulatory network. Building on tracer injection and Doppler ultrasound studies, computational simulations have elucidated how the fetal cardiovascular system changes throughout gestation under healthy and pathological conditions. Reduced-order LPN models of fetal circulation are the most utilized representation of fetal circulation to date. LPNs have been used to describe blood flow distribution at different gestational stages and uncovered the systemic effect of specific hemodynamic alterations seen in postnatal transition and CHDs. Computational models enable a non-invasive measurement of fetal blood pressure and vascular resistance, lending new mechanistic insights into various fetal pathologies and enabling more accurate patient stratification schemes for feto-maternal diseases. Three-dimensional (3D) blood flow simulations are needed to elucidate complex local flow patterns within the fetal heart and vessels of interest. in silico modifications of vessel geometries link the unique morphologies of fetal-specific vessels, such as the ductus venosus and umbilical arteries, to their function. When these in silico perturbations are made to approximate CHDs, resulting phenotypes offer insight into morphological and hemodynamic interdependence.

Existing computational studies of fetal hemodynamics are not without limitations. LPNs neglect the spatial heterogeneity of velocity and pressure fields in a vessel, and they assume a Poiseuille flow profile that is not representative of vessels with complex morphologies such as the umbilical arteries and the ductus venosus. These assumptions lead to inaccuracies in pressure and flow rate measurements, and they obscure spatially resolved hemodynamic stress maps that can localize vascular remodeling or disease progression [17,176]. Allometric scaling principles assume that every part of the fetal body grows in a uniform manner, which may not hold in diseased cases. For example, asymmetric growth restriction leads the lower fetal body to be more severely underdeveloped than the head [58], so brain vessels could be larger than allometric principles would predict. The existing 3D simulation studies often leverage idealized geometries and rigid wall models. Idealized geometries neglect the irregularities and variation seen in population dynamics. The impact of these intricacies on hemodynamics should be more extensively characterized.

In addition to the immediate hemodynamic impacts captured by the in silico perturbations presented throughout this paper, it is also important to study vessel maladaption from its infancy. Cardiovascular defects often arise from the abnormal remodeling of earlier embryonic structures (the heart tube, pharyngeal arch arteries, cardinal veins, etc.), occuring in much earlier gestational stages than those investigated by most computational studies of human fetal circulation. Since the chick embryos are accessible from an early gestation and their cardiovascular development mirrors that of humans, a vast body of literature is dedicated to uncovering the mechanism behind the emergence of CHD phenotypes using the early chick embryo through both experiments and computational hemodynamics simulations [32,36,38].

The validation of fetal hemodynamic simulation results is key to their accuracy and reliability. As with pediatric and adult simulations, fetal simulation results are typically validated using in vitro or in vivo (Doppler) measurements and comparisons [133,134,135,136,141,142,170]. While Doppler measurements are excellent for reduced-order model validation, 3D simulations may benefit from more extensive spatial validation. The spatial features of an in silico velocity field can be validated using in vitro phantoms or in vivo 4D MRI measurements [27,177,178,179,180], which will become possible for fetal circulation studies as fetal MRI technologies continue to advance. Validation can be particularly challenging for studies with experimentally inaccessible parameters. In such cases, investigators may consider extensive characterization of the numerical method used for parameter estimation, the use of a surrogate model containing synthetic data, or incorporating uncertainty quantification to inform the confidence in the estimated parameter value [181,182].

As it stands, the breadth of modeling-based studies of human fetal circulation remains rather limited. The mechanical principles regulating the interaction and distribution of fetal blood flow streams are yet to be systemically reported. For example, the supply of oxygenated blood to the left heart relies heavily on the complex flow environment in the inferior vena cava and right ventricle, where multiple blood streams of drastically different oxygen saturation converge. To what degrees are the streams mixing? What is maintaining their limited mixing and diversion? Can the flows be destabilized with perturbations to the venous vascular system, and what are the functional consequences? Answering these questions can be critical in uncovering the underlying principles of the function of the fetal vascular system, which warrants the requisite detailed quantitative studies of the fluid mechanics and convective oxygen transport in the fetal venous system. LPN simulations can be adapted to study the hemodynamic and developmental impacts of a broader class of fetal cardiovascular anomalies such as HLHS and TOF. Adding oxygen transport models has the potential to greatly deepen our understanding of these diseases. Fetal LPN models may also be integrated with a systemic model of maternal fetal circulation. A combined description of fetal-materno circulation paves the way for a quantitative, comprehensive understanding of pre-eclampsia and other conditions where maternal cardiovascular complications impact fetal health. Additionally, while the existing LPN models of fetal circulation show some level of variability in their construction, the impact of LPN model topology on flow waveform calculation or parameter estimation has not been investigated.

**Figure 8:**
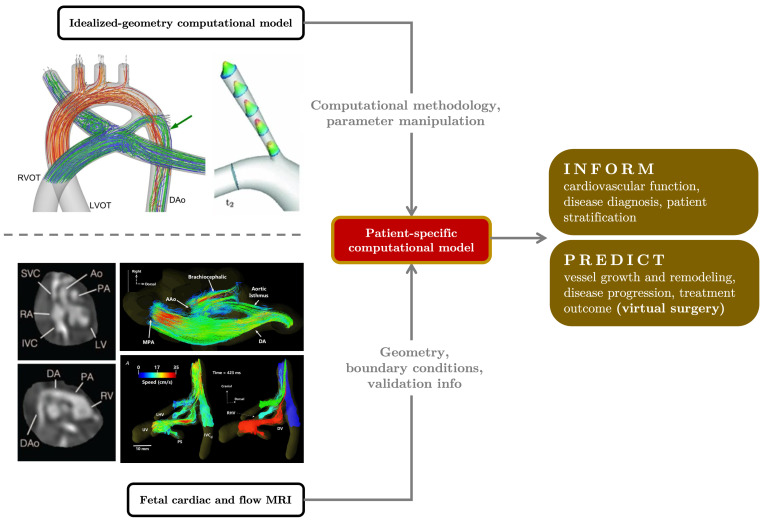
Outlook for patient-specific fetal hemodynamic modeling. Vascular morphology and flow information provided by fetal MRI ([84,183]) can be integrated with existing computational frameworks established using idealized geometry models (examples from [27,170]) to create patient-specific models of fetal circulation. The detailed blood flow information and predicative capability provided by computational modeling provide patient-specific insights that can drive physiology studies, disease characterizations, and clinical decision making.

Achieving patient-specificity in the computational modeling of fetal hemodynamics is critical for its adoption to clinical practices (Figure 8). The vascular system of each individual displays unique features, and the way a CHD manifests in one individual can be drastically different from that in another. It is therefore sensible to question how well an idealized model represents the population. Optimal treatment plans for two patients likely depend on their individual vascular characteristics. In order for virtual surgery planning to be adopted clinically, each model must accurately represent a patient’s vascular system to reliably predict the efficacy to a particular intervention. Multiscale simulations in which a 3D anatomical model is connected to an LPN of the rest of circulation offer the benefit of capturing local hemodynamics in a region of interest and its effect on various organs. Fetal MRI technology can enable more multiscale simulations of fetal circulation, ushering in a new frontier of human fetal circulation studies. Improved geometric resolution allows for vessel shape quantification among healthy individuals and CHD patients. High-fidelity volumetric vessel images enable the construction of patient-specific vascular models that are necessary for patient-specific 3D simulations. Flow information obtained using PC-MRI or 4D flow MRI can be used to validate 3D simulation results. Similarly, oxygen saturation levels measured using MRI will aid method development efforts for the simulation of oxygen transport in fetal circulation. Computational simulations have greatly contributed to current knowledge surrounding human fetal circulation. With new technology, these simulations may become even more informative, ushering in a new era for fetal health and interventions.

## Figures and Tables

**Table 1:** Mean blood pressure and flow rate in human fetal organ-specific capillary networks.

	Mean Pressure (mmHg)	Flow Rate (%CCO) ^a^
Source	Yigit et al., 2015 [78]	Abduljalil et al., 2021 [73]
Methodology	Simulated using LPN	Compiled from ultrasound and MRI data
Gestational Age ^b^	40 weeks	10≤t≤40 weeks
Sample Size	-	Varies
Brain	32	$4.692{\left(t-2\right)}^{0.3618}\;\;\left(n=134\right)$
Lungs	13	22.0 (n=375)
Upper Limbs	19	-
Liver	6	$2.640\times 10^{-4}t^{3}-0.02869t^{2}+0.7891t-14.09\;\;\left(n=558\right)$
Intestine	12	-
Kidney	34	10.57−0.1238t(n=103)
Placenta	26	33.34−0.4391t(n=1394)

^a^ %CCO: Percentage of combined cardiac output. Abduljalil et al., 2021 [73] report CCO (mL/min) as a double exponential functions of gestational age (*t*, in weeks) as $\mathrm{CCO}\left(t\right)=3400.88\times {\left(1.141\times 10^{-5}\right)}^{e^{-0.07022(t-2)}}\;\;\left(n=656\right)$. ^b^ Gestational age = fetal age + 2.

**Table 2:** Oxygen concentration and blood flow rate in major vessels of healthy human fetuses measured using MRI. Data presented as mean ± SD.

	Oxygen Saturation (%)	Flow Rate (%CCO)
Source	Saini et al., 2020 [89]	Prsa et al., 2014 [88]
Methodology	T2-MRI Oximetry	PC-MRI
Gestational Age	37.0±1.1 weeks	36±1 weeks
Sample Size	40 fetuses	30 fetuses
Ascending Aorta	68±10	41±6
Main Pulmonary Artery	49±9	56±6
Superior Vena Cava	-	29±7
Ductus Arteriosus	-	40±8
Descending Aorta	57±10	55±10
Umbilical Vein	85±9	29±9
Foramen Ovale	-	29±11

**Table 3:** Analogous circuit elements used in lumped-parameter hemodynamics models and the flow phenomenon they capture.

Circuit Element	Symbol	Hemodynamic Interpretation	Used for
Linear Resistor	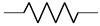	Resistance to viscous flow	Vessels, peripheral vascular beds, heart chambers.
Nonlinear Resistor	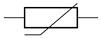	Energy dissipation due to irregular local hemodynamics	Heart valves, vessels with abrupt changes in diameter (e.g., stenosis), etc.
Capacitor	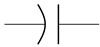	Changes in flow due to vessel expansion and contraction	Compliant vessels and peripheral vascular beds.
Variable Capacitor	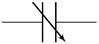	Time-varying changes in myocardial compliance	Contracting heart chambers.
Inductor	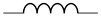	Inertia of flowing blood	Large arteries, heart chambers.
Diode	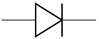	Unidirectional flow	Heart valves.
Current Source	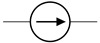	Prescribed flow rate waveform	Inlets with available Doppler measurements.

**Table 4:** Hemodynamic indices of the heart chambers and major vessels of healthy, full-term human fetuses obtained from LPNs [78].

	Mean Pressure (mmHg)	Max Pressure (mmHg)	Min Pressure (mmHg)	Flow Rate (%CCO)
Left Atrium	3.0	4.0	1.5	57
Left Ventricle	25.0	55.0	1.9	57
Right Atrium	3.5	4.9	1.9	43
Right Ventricle	26.0	57.0	2.0	43
Foramen Ovale	-	-	-	21
Ascending Aorta	45.0	53.0	36.0	43
Carotid Arteries	42.0	48.0	35.0	21
Descending Aorta	42.0	48.0	36.0	47
Main Pulmonary Artery	46.0	54.0	37.0	57
Ductus Arteriosus	-	-	-	35
Umbilical Arteries (Fetal End)	41.0	47.0	35.0	22
Umbilical Vein (Placental End)	8.0	-	-	22
Ductus Venosus	-	-	-	8
Thoracic Inferior Vena Cava	4.9	5.5	4.4	47
Superior Vena Cava	5.0	5.5	4.8	31

## Data Availability

Not applicable.

## Abbreviations

The following abbreviations are used in this manuscript:

3D	Three-dimensional
4D	Four-dimensional
BC	Boundary condition
CCO	Combined cardiac output
CHD	Congenital heart defect
DCC	Delayed cord clamping
FSI	Fluid–structure interaction
FMDM	Fetus of mother with diabetes mellitus
HLHS	Hypoplastic left heart syndrome
ICC	Immediate cord clamping
IUGR	Intrauterine growth restriction
LPN	Lumped parameter network
MRI	Magnetic resonance imaging
PC-MRI	Phase contrast magnetic resonance imaging
TOF	Tetralogy of Fallot

## Appendix A. Oxygen Saturation in the Fetal Cardiovascular System

**Table A1:** Oxygen Saturation in the heart chambers and major vessels of healthy, full-term human fetuses obtained from LPNs [78].

	Oxygen Saturation (%)		Oxygen Saturation (%)
Left Atrium	49	Main Pulmonary Artery	42
Left Ventricle	49	Ductus Arteriosus	42
Right Atrium	42	Umbilical Arteries (Fetal End)	43.7
Right Ventricle	42	Umbilical Vein (Placental End)	69.4
Foramen Ovale	-	Ductus Venosus	69.4
Ascending Aorta	49	Thoracic Inferior Vena Cava	49.9
Carotid Arteries	49		
Descending Aorta	43.7	Superior Vena Cava	40.2

## Appendix B. Characteristic Scales of Fetal Blood Flow

The Reynolds number (Re) is calculated using the maximum velocity $v_{\max}$ of blood flow across a valve or in a vessel and the diameter of the valve or vessel *D* as

(A1) $$Re=\frac{\rho v_{\max}D}{\mu },$$

with ρ=1.06 g cm${}^{-3}$ and μ=0.04 dyne s cm${}^{-2}$ being the density and dynamic viscosity of blood. Median or mean peak velocity ($v_{\max}$) and diameter (*D*) values are used when calculating the average Reynolds number per cohort.

**Table A2:** Velocity and length scale in the fetal heart and vessels and the associated Reynolds number. Note that laminar flow conditions generally prevail in blood vessels with Reynolds numbers as high as 10,000.

	Gestational Week	Peak Velocity (cm/s)	Diameter (cm)	Reynolds Number
Mitral Valve ^1^	21	41	0.49	532
	27	45	0.71	847
	35	47	0.93	1158
Tricuspic Valve ^2^	21	45	0.49	584
	27	49	0.74	961
	35	51	1.00	1352
Ascending Aorta ^3^	20	55.6	0.33	487
	24	71.0	0.43	809
	28	84.8	0.53	1191
	32	96.3	0.64	1633
	36	104.7	0.73	2026
	40	109.7	0.83	2413
Main Pulmonary Artery ^4^	20	55.1	0.36	526
	24	64.6	0.48	822
	28	73.6	0.60	1170
	32	80.7	0.71	1518
	36	86.2	0.82	1873
	40	88.9	0.93	2190
Ductus Arteriosus ^5^	22	75.9	0.238	479
	26	92.0	0.270	658
	30	107	0.304	862
	34	127	0.349	1175
Umbilical Artery ^6^	20	41.05	0.252	274
	24	45.47	0.341	411
	28	48.92	0.402	521
	32	51.65	0.433	593
	36	53.93	0.447	639
	40	55.80	0.464	686
Ductus Venosus ^7^	20	49.3	0.143	187
	24	55.7	0.152	224
	28	60.8	0.160	258
	32	62.8	0.168	280
	36	61.1	0.177	287
	40	55.2	0.189	276

^1^ N = 307 subjects, peak velocity (Doppler) [62]; N = 159 subjects, diameter (ultrasound measurements) [67]. ^2^ N = 258 subjects, peak velocity (Doppler) [62]; N = 161 subjects, diameter (ultrasound measurements) [67]. ^3^ N = 926 subjects, peak velocity (Doppler) [63]; N = 296 subjects, diameter (ultrasund measuremnts) [67]. ^4^ N = 222 subjects, peak velocity (Doppler) [64]; N = 312 subjects, diameter (ultrasound measurements) [67]. ^5^ N = 222 subjects, peak velocity (Doppler) [64]; N = 131 subjects, diameter (ultrasound measurements) [68]. ^6^ N = 133 subjects, peak velocity (Doppler) [65]; N = 2310 subjects, diameter (ultrasound measurements) [69]. ^7^ N = 230 subjects, peak velocity (Doppler) [66]; N = 230 subjects, diameter (ultrasound measurements) [66].

## References

[1] Rana M.S., Sizarov A., Christoffels V.M., Moorman A.F. (2014). Development of the human aortic arch system captured in an interactive three-dimensional reference model. Am. J. Med. Genet. Part A.

[2] Yagel S., Kivilevitch Z., Cohen S.M., Valsky D.V., Messing B., Shen O., Achiron R. (2010). The fetal venous system, Part I: Normal embryology, anatomy, hemodynamics, ultrasound evaluation and Doppler investigation. Ultrasound Obstet. Gynecol..

[3] Yee K., Lui F. (2021). Anatomy, Thorax, Heart Foramen Ovale. StatPearls [Internet].

[4] Lindsey S.E., Butcher J.T., Yalcin H.C. (2014). Mechanical regulation of cardiac development. Front. Physiol..

[5] Chew L.C., Verma R.P. (2021). Fetal Growth Restriction.

[6] Donofrio M., Bremer Y., Schieken R., Gennings C., Morton L., Eidem B., Cetta F., Falkensammer C., Huhta J., Kleinman C. (2003). Autoregulation of cerebral blood flow in fetuses with congenital heart disease: The brain sparing effect. Pediatr. Cardiol..

[7] Itsukaichi M., Kikuchi A., Yoshihara K., Serikawa T., Takakuwa K., Tanaka K. (2011). Changes in Fetal Circulation Associated with Congenital Heart Disease and Their Effects on Fetal Growth. Fetal Diagn. Ther..

[8] Krishna U., Bhalerao S. (2011). Placental insufficiency and fetal growth restriction. J. Obstet. Gynecol. India.

[9] Miller S.L., Huppi P.S., Mallard C. (2016). The consequences of fetal growth restriction on brain structure and neurodevelopmental outcome. J. Physiol..

[10] McBride K.L., Zender G.A., Fitzgerald-Butt S.M., Koehler D., Menesses-Diaz A., Fernbach S., Lee K., Towbin J.A., Leal S., Belmont J.W. (2009). Linkage analysis of left ventricular outflow tract malformations (aortic valve stenosis, coarctation of the aorta, and hypoplastic left heart syndrome). Eur. J. Hum. Genet..

[11] Kiserud T., Acharya G. (2004). The fetal circulation. Prenat. Diagn..

[12] Kiserud T. (2005). Physiology of the fetal circulation. Semin. Fetal Neonatal Med..

[13] Lasheras J.C. (2007). The biomechanics of arterial aneurysms. Annu. Rev. Fluid Mech..

[14] Kanter K.R., Haggerty C.M., Restrepo M., de Zelicourt D.A., Rossignac J., Parks W.J., Yoganathan A.P. (2012). Preliminary clinical experience with a bifurcated Y-graft Fontan procedure—A feasibility study. J. Thorac. Cardiovasc. Surg..

[15] Taylor C.A., Fonte T.A., Min J.K. (2013). Computational Fluid Dynamics Applied to Cardiac Computed Tomography for Noninvasive Quantification of Fractional Flow Reserve. J. Am. Coll. Cardiol..

[16] Poelma C., Watton P.N., Ventikos Y. (2015). Transitional flow in aneurysms and the computation of haemodynamic parameters. J. R. Soc. Interface.

[17] Grande Gutiérrez N., Mathew M., McCrindle B.W., Tran J.S., Kahn A.M., Burns J.C., Marsden A.L. (2019). Hemodynamic variables in aneurysms are associated with thrombotic risk in children with Kawasaki disease. Int. J. Cardiol..

[18] Schwarz E.L., Kelly J.M., Blum K.M., Hor K.N., Yates A.R., Zbinden J.C., Verma A., Lindsey S.E., Ramachandra A.B., Szafron J.M. (2021). Hemodynamic performance of tissue-engineered vascular grafts in Fontan patients. NPJ Regen. Med..

[19] Taylor C.A., Draney M.T., Ku J.P., Parker D., Steele B.N., Wang K., Zarins C.K. (1999). Predictive medicine: Computational techniques in therapeutic decision-making. Comput. Aided Surg..

[20] Gundert T.J., Marsden A.L., Yang W., LaDisa J.F. (2012). Optimization of Cardiovascular Stent Design Using Computational Fluid Dynamics. J. Biomech. Eng..

[21] Lindsey S.E., Menon P.G., Kowalski W.J., Shekhar A., Yalcin H.C., Nishimura N., Schaffer C.B., Butcher J.T., Pekkan K. (2015). Growth and hemodynamics after early embryonic aortic arch occlusion. Biomech. Model. Mechanobiol..

[22] Lashkarinia S.S., Piskin S., Bozkaya T.A., Salihoglu E., Yerebakan C., Pekkan K. (2018). Computational pre-surgical planning of arterial patch reconstruction: Parametric limits and in vitro validation. Ann. Biomed. Eng..

[23] Anbazhakan S., Rios Coronado P.E., Sy-Quia A.N.L., Seow L.W., Hands A.M., Zhao M., Dong M.L., Pfaller M.R., Amir Z.A., Raftrey B.C. (2022). Blood flow modeling reveals improved collateral artery performance during the regenerative period in mammalian hearts. Nat. Cardiovasc. Res..

[24] Garcia-Cañadilla P., Rudenick P.A., Crispi F., Cruz-Lemini M., Palau G., Camara O., Gratacos E., Bijens B.H. (2014). A Computational Model of the Fetal Circulation to Quantify Blood Redistribution in Intrauterine Growth Restriction. PLoS Comput. Biol..

[25] Wong H.S., Wiputra H., Tulzer A., Tulzer G., Yap C.H. (2022). Fluid Mechanics of Fetal Left Ventricle During Aortic Stenosis with Evolving Hypoplastic Left Heart Syndrome. Ann. Biomed. Eng..

[26] Wiputra H., Chen C.K., Talbi E., Lim G.L., Soomar S.M., Biswas A., Mattar C.N.Z., Bark D., Leo H.L., Yap C.H. (2018). Human fetal hearts with tetralogy of Fallot have altered fluid dynamics and forces. Am. J. Physiol.-Heart Circ. Physiol..

[27] Pekkan K., Dasi L.P., Nourparvar P., Yerneni S., Tobita K., Fogel M.A., Keller B., Yoganathan A. (2008). In vitro hemodynamic investigation of the embryonic aortic arch at late gestation. J. Biomech..

[28] Langille B.L. (1996). Arterial remodeling: Relation to hemodynamics. Can. J. Physiol. Pharmacol..

[29] Sedmera D., Hu N., Weiss K.M., Keller B.B., Denslow S., Thompson R.P. (2002). Cellular changes in experimental left heart hypoplasia. Anat. Rec..

[30] Li Y.S.J., Haga J.H., Chien S. (2005). Molecular basis of the effects of shear stress on vascular endothelial cells. J. Biomech..

[31] Lindsey S.E., Butcher J.T., Vignon-Clementel I.E. (2018). Cohort-based multiscale analysis of hemodynamic-driven growth and remodeling of the embryonic pharyngeal arch arteries. Development.

[32] Salman H.E., Alser M., Shekhar A., Gould R.A., Benslimane F.M., Butcher J.T., Yalcin H.C. (2021). Effect of left atrial ligation-driven altered inflow hemodynamics on embryonic heart development: Clues for prenatal progression of hypoplastic left heart syndrome. Biomech. Model. Mechanobiol..

[33] Swanson A., David A. (2015). Animal models of fetal growth restriction: Considerations for translational medicine. Placenta.

[34] Everett N.B., Johnson R.J. (1950). Use of Radioactive Phosphorus in Studies of Fetal Circulation. Am. J. Physiol.-Leg. Content.

[35] Zhou Y.Q., Cahill L.S., Wong M.D., Seed M., Macgowan C.K., Sled J.G. (2014). Assessment of flow distribution in the mouse fetal circulation at late gestation by high-frequency Doppler ultrasound. Physiol. Genom..

[36] Salman H.E., Yalcin H.C. (2021). Computational Modeling of Blood Flow Hemodynamics for Biomechanical Investigation of Cardiac Development and Disease. J. Cardiovasc. Dev. Dis..

[37] Kowalski W.J., Teslovich N.C., Menon P.G., Tinney J.P., Keller B.B., Pekkan K. (2014). Left atrial ligation alters intracardiac flow patterns and the biomechanical landscape in the chick embryo. Dev. Dyn..

[38] Lindsey S.E., Vignon-Clementel I.E., Butcher J.T. (2021). Assessing early cardiac outflow tract adaptive responses through combined experimental-computational manipulations. Ann. Biomed. Eng..

[39] Jensen O.E., Chernyavsky I.L. (2019). Blood Flow and Transport in the Human Placenta. Annu. Rev. Fluid Mech..

[40] Kiserud T. (2001). The ductus venosus. Semin. Perinatol..

[41] Markl M., Frydrychowicz A., Kozerke S., Hope M., Wieben O. (2012). 4D flow MRI. J. Magn. Reson. Imaging.

[42] Sun L., Macgowan C.K., Portnoy S., Sled J.G., Yoo S.J., Grosse-Wortmann L., Jaeggi E., Kingdom J., Seed M. (2017). New advances in fetal cardiovascular magnetic resonance imaging for quantifying the distribution of blood flow and oxygen transport: Potential applications in fetal cardiovascular disease diagnosis and therapy. Echocardiography.

[43] Mielke G., Benda N. (2001). Cardiac Output and Central Distribution of Blood Flow in the Human Fetus. Circulation.

[44] Murphy P.J. (2005). The fetal circulation. Contin. Educ. Anaesth. Crit. Care Pain.

[45] Strong T.H., Jarles D.L., Vega J.S., Feldman D.B. (1994). The umbilical coiling index. Am. J. Obstet. Gynecol..

[46] Predanic M., Perni S.C., Chervenak F.A. (2006). Antenatal umbilical coiling index and Doppler flow characteristics. Ultrasound Obstet. Gynecol..

[47] Sharma B., Bhardwaj N., Gupta S., Gupta P.K., Verma A., Malviya K. (2012). Association of umbilical coiling index by colour Doppler ultrasonography at 18–22 weeks of gestation and perinatal outcome. J. Obstet. Gynecol. India.

[48] Devaru D., Thusoo M. (2012). Umbilical coiling index & the perinatal outcome. J. Obstet. Gynecol. India.

[49] Pergialiotis V., Kotrogianni P., Koutaki D., Christopoulos-Timogiannakis E., Papantoniou N., Daskalakis G. (2020). Umbilical cord coiling index for the prediction of adverse pregnancy outcomes: A meta-analysis and sequential analysis. J. Matern.-Fetal Neonatal Med..

[50] Peng H.Q., Smith-Levitin M., Rochelson B., Kahn E. (2006). Umbilical cord stricture and overcoiling are common causes of fetal demise. Pediatr. Dev. Pathol..

[51] Gilbert R.D., Schroder H., Kawamura T., Dale P.S., Power G.G. (1985). Heat transfer pathways between fetal lamb and ewe. J. Appl. Physiol..

[52] Schrauben E.M., Saini B.S., Darby J.R., Soo J.Y., Lock M.C., Stirrat E., Stortz G., Sled J.G., Morrison J.L., Seed M. (2019). Fetal hemodynamics and cardiac streaming assessed by 4D flow cardiovascular magnetic resonance in fetal sheep. J. Cardiovasc. Magn. Reson..

[53] Finnemore A., Groves A. (2015). Physiology of the fetal and transitional circulation. Semin. Fetal Neonatal Med..

[54] Mott J.C. (1982). Control of the foetal circulation. J. Exp. Biol..

[55] Nuwayhid B., Brinkman C., Su C., Bevan J., Assali N. (1975). Development of autonomic control of fetal circulation. Am. J. Physiol.-Leg. Content.

[56] Cohen E., Baerts W., van Bel F. (2015). Brain-sparing in intrauterine growth restriction: Considerations for the neonatologist. Neonatology.

[57] Baschat A.A. (2006). The fetal circulation and essential organs—a new twist to an old tale. Ultrasound Obstet. Gynecol..

[58] Peleg D., Kennedy C.M., Hunter S.K. (1998). Intrauterine growth restriction: Identification and management. Am. Fam. Physician.

[59] Pohlman A.G. (1909). The course of the blood through the heart of the fetal mammal, with a note on the reptilian and amphibian circulations. Anat. Rec..

[60] Rudolph A.M., Heymann M.A. (1967). The Circulation of the Fetus in Utero. Circ. Res..

[61] Barclay A.E., Barcroft J., Barron D.H., Franklin K.J. (1939). A Radiographic Demonstration of the Circulation through the Heart in the Adult and in the Fœtus, and the Identification of the Ductus Arteriosus. Br. J. Radiol..

[62] Harada K., Rice M.J., Shiota T., Ishii M., McDonald R.W., Reller M.D., Sahn D.J. (1997). Gestational age-and growth-related alterations in fetal right and left ventricular diastolic filling patterns. Am. J. Cardiol..

[63] Bahlmann F., Wellek S., Reinhardt I., Krummenauer F., Merz E., Welter C. (2001). Reference values of fetal aortic flow velocity waveforms and associated intra-observer reliability in normal pregnancies. Ultrasound Obstet. Gynecol. Off. J. Int. Soc. Ultrasound Obstet. Gynecol..

[64] Mielke G., Benda N. (2000). Blood flow velocity waveforms of the fetal pulmonary artery and the ductus arteriosus: Reference ranges from 13 weeks to term. Ultrasound Obstet. Gynecol..

[65] Acharya G., Wilsgaard T., Berntsen G., Maltau J., Kiserud T. (2005). Reference ranges for serial measurements of blood velocity and pulsatility index at the intra-abdominal portion, and fetal and placental ends of the umbilical artery. Ultrasound Obstet. Gynecol. Off. J. Int. Soc. Ultrasound Obstet. Gynecol..

[66] Zytoon A.A., El-Abakawy N.N.A., Hassanein S.A.h. (2020). Reference values for ductus venosus flow in normal gestation among an Egyptian population. Egypt. J. Radiol. Nucl. Med..

[67] Sharland G., Allan L. (1992). Normal fetal cardiac measurements derived by cross-sectional echocardiography. Ultrasound Obstet. Gynecol. Off. J. Int. Soc. Ultrasound Obstet. Gynecol..

[68] Szpinda M., Szwesta A., Szpinda E. (2007). Morphometric study of the ductus arteriosus during human development. Ann. Anat.-Anat. Anz..

[69] Barbieri C., Cecatti J., Surita F., Marussi E., Costa J. (2012). Sonographic measurement of the umbilical cord area and the diameters of its vessels during pregnancy. J. Obstet. Gynaecol..

[70] FitzGerald D.E., Drumm J.E. (1977). Non-invasive measurement of human fetal circulation using ultrasound: A new method. BMJ.

[71] Campbell S., Vyas S., Nicolaides K.H. (1991). Doppler investigation of the fetal circulation. J. Perinat. Med..

[72] Molina F., Faro C., Sotiriadis A., Dagklis T., Nicolaides K. (2008). Heart stroke volume and cardiac output by four-dimensional ultrasound in normal fetuses. Ultrasound Obstet. Gynecol..

[73] Abduljalil K., Pan X., Clayton R., Johnson T.N., Jamei M. (2021). Fetal physiologically based pharmacokinetic models: Systems information on fetal cardiac output and its distribution to different organs during development. Clin. Pharmacokinet..

[74] Hecher K., Campbell S., Doyle P., Harrington K., Nicolaides K. (1995). Assessment of Fetal Compromise by Doppler Ultrasound Investigation of the Fetal Circulation. Circulation.

[75] Harrington K., Carpenter R.G., Nguyen M., Campbell S. (1995). Changes observed in Doppler studies of the fetal circulation in pregnancies complicated by pre-eclampsia or the delivery of a small-for-gestational-age baby. I. Cross-sectional analysis. Ultrasound Obstet. Gynecol..

[76] Harrington K., Thompson M.O., Carpenter R.G., Nguyen M., Campbell S. (1999). Doppler fetal circulation in pregnancies complicated by pre-eclampsia or delivery of a small for gestational age baby: 2. Longitudinal analysis. BJOG Int. J. Obstet. Gynaecol..

[77] Hecher K., Ville Y., Snijders R., Nicolaides K. (1995). Doppler studies of the fetal circulation in twin–twin transfusion syndrome. Ultrasound Obstet. Gynecol..

[78] (2015). Transition from fetal to neonatal circulation: Modeling the effect of umbilical cord clamping. J. Biomech..

[79] Ranke C., Hendrickx P., Roth U., Brassel F., Creutzig A., Alexander K. (1992). Color and conventional image-directed Doppler ultrasonography: Accuracy and sources of error in quantitative blood flow measurements. J. Clin. Ultrasound.

[80] Saleem S.N. (2008). Feasibility of MRI of the Fetal Heart with Balanced Steady-State Free Precession Sequence Along Fetal Body and Cardiac Planes. Am. J. Roentgenol..

[81] Dashe J.S., McIntire D.D., Twickler D.M. (2009). Maternal obesity limits the ultrasound evaluation of fetal anatomy. J. Ultrasound Med..

[82] Lloyd D.F., Pushparajah K., Simpson J.M., Van Amerom J.F., Van Poppel M.P., Schulz A., Kainz B., Deprez M., Lohezic M., Allsop J. (2019). Three-dimensional visualisation of the fetal heart using prenatal MRI with motion-corrected slice-volume registration: A prospective, single-centre cohort study. Lancet.

[83] van Amerom J.F., Lloyd D.F., Deprez M., Price A.N., Malik S.J., Pushparajah K., van Poppel M.P., Rutherford M.A., Razavi R., Hajnal J.V. (2019). Fetal whole-heart 4D imaging using motion-corrected multi-planar real-time MRI. Magn. Reson. Med..

[84] Roy C.W., van Amerom J.F., Marini D., Seed M., Macgowan C.K. (2019). Fetal cardiac MRI: A review of technical advancements. Top. Magn. Reson. Imaging.

[85] Sun L., Marini D., Saini B., Schrauben E., Macgowan C.K., Seed M. (2020). Understanding Fetal Hemodynamics Using Cardiovascular Magnetic Resonance Imaging. Fetal Diagn. Ther..

[86] Seed M., van Amerom J.F.P., Yoo S.J., Bahiyah Al Nafisi L.G.W., Jaeggi E., Jansz M.S., Macgowan C.K. (2012). Feasibility of quantification of the distribution of blood flow in the normal human fetal circulation using CMR: A cross-sectional study. J. Cardiovasc. Magn. Reson..

[87] Goolaub D.S., Xu J., Schrauben E.M., Marini D., Kingdom J.C., Sled J.G., Seed M., Macgowan C.K. (2022). Volumetric Fetal Flow Imaging with Magnetic Resonance Imaging. IEEE Trans. Med. Imaging.

[88] Prsa M., Sun L., van Amerom J., Yoo S.J., Grosse-Wortmann L., Jaeggi E., Macgowan C., Seed M. (2014). Reference Ranges of Blood Flow in the Major Vessels of the Normal Human Fetal Circulation at Term by Phase-Contrast Magnetic Resonance Imaging. Circ. Cardiovasc. Imaging.

[89] Saini B.S., Darby J.R., Portnoy S., Sun L., van Amerom J., Lock M.C., Soo J.Y., Holman S.L., Perumal S.R., Kingdom J.C. (2020). Normal human and sheep fetal vessel oxygen saturations by T2 magnetic resonance imaging. J. Physiol..

[90] Batchelor G.K. (2000). An Introduction to Fluid Dynamics.

[91] Secomb T.W. (2017). Blood Flow in the Microcirculation. Annu. Rev. Fluid Mech..

[92] Al-Roubaie S., Jahnsen E.D., Mohammed M., Henderson-Toth C., Jones E.A.V. (2011). Rheology of embryonic avian blood. Am. J. Physiol.-Heart Circ. Physiol..

[93] Gonzalo A., García-Villalba M., Rossini L., Durán E., Vigneault D., Martínez-Legazpi P., Flores O., Bermejo J., McVeigh E., Kahn A.M. (2022). Non-Newtonian blood rheology impacts left atrial stasis in patient-specific simulations. Int. J. Numer. Methods Biomed. Eng..

[94] Reymond P., Crosetto P., Deparis S., Quarteroni A., Stergiopulos N. (2013). Physiological simulation of blood flow in the aorta: Comparison of hemodynamic indices as predicted by 3-D FSI, 3-D rigid wall and 1-D models. Med. Eng. Phys..

[95] Sengupta D., Kahn A.M., Kung E., Esmaily Moghadam M., Shirinsky O., Lyskina G.A., Burns J.C., Marsden A.L. (2014). Thrombotic risk stratification using computational modeling in patients with coronary artery aneurysms following Kawasaki disease. Biomech. Model. Mechanobiol..

[96] Chen H.Y., Zhu L., Huo Y., Liu Y., Kassab G.S. (2010). Fluid–structure interaction (FSI) modeling in the cardiovascular system. Computational Cardiovascular Mechanics.

[97] Hirschhorn M., Tchantchaleishvili V., Stevens R., Rossano J., Throckmorton A. (2020). Fluid–structure interaction modeling in cardiovascular medicine – A systematic review 2017–2019. Med Eng. Phys..

[98] Hughes T.J.R., Liu W.K., Zimmermann T.K. (1981). Lagrangian-Eulerian finite element formulation for incompressible viscous flows. Comput. Methods Appl. Mech. Eng..

[99] Figueroa C.A., Vignon-Clementel I.E., Jansen K.E., Hughes T.J.R., Taylor C.A. (2006). A coupled momentum method for modeling blood flow in three-dimensional deformable arteries. Comput. Methods Appl. Mech. Eng..

[100] Womersley J.R. (1957). An Elastic Tube Theory of Pulse Transmission and Oscillatory Flow in Mammalian Arteries.

[101] Cheynet E. (2021). Pcolor in Polar Coordinates: Version 3.11. https://www.mathworks.com/matlabcentral/fileexchange/49040-pcolor-in-polar-coordinates.

[102] Alastruey J., Siggers J.H., Peiffer V., Doorly D.J., Sherwin S.J. (2012). Reducing the data: Analysis of the role of vascular geometry on blood flow patterns in curved vessels. Phys. Fluids.

[103] Larsson S., Thomée V. (2003). Partial Differential Equations with Numerical Methods.

[104] Pfaller M.R., Pham J., Verma A., Pegolotti L., Wilson N.M., Parker D.W., Yang W., Marsden A.L. (2022). Automated generation of 0D and 1D reduced-order models of patient-specific blood flow. Int. J. Numer. Methods Biomed. Eng..

[105] Wilson N.M., Arko F.R., Taylor C.A. (2005). Predicting changes in blood flow in patient-specific operative plans for treating aortoiliac occlusive disease. Comput. Aided Surg..

[106] Updegrove A., Wilson N.M., Merkow J., Lan H., Marsden A.L., Shadden S.C. (2017). SimVascular: An open source pipeline for cardiovascular simulation. Ann. Biomed. Eng..

[107] Vignon-Clementel I.E., Figueroa C.A., Jansen K.E., Taylor C.A. (2006). Outflow boundary conditions for three-dimensional finite element modeling of blood flow and pressure in arteries. Comput. Methods Appl. Mech. Eng..

[108] Vignon-Clementel I.E., Figueroa C.A., Jansen K.E., Taylor C.A. (2010). Outflow boundary conditions for 3D simulations of non-periodic blood flow and pressure fields in deformable arteries. Comput. Methods Biomech. Biomed. Eng..

[109] Coogan J.S., Humphrey J.D., Figueroa C.A. (2013). Computational simulations of hemodynamic changes within thoracic, coronary, and cerebral arteries following early wall remodeling in response to distal aortic coarctation. Biomech. Model. Mechanobiol..

[110] Sengupta D., Kahn A.M., Burns J.C., Sankaran S., Shadden S.C., Marsden A.L. (2012). Image-based modeling of hemodynamics in coronary artery aneurysms caused by Kawasaki disease. Biomech. Model. Mechanobiol..

[111] Miliić V., Quarteroni A. (2004). Analysis of lumped parameter models for blood flow simulations and their relation with 1D models. Esaim Math. Model. Numer. Anal..

[112] Karimi A., Sera T., Kudo S., Navidbakhsh M. (2016). Experimental verification of the healthy and atherosclerotic coronary arteries incompressibility via Digital Image Correlation. Artery Res..

[113] Pennati G., Migliavacca F., Dubini G., Pietrabissa R., de Leval M.R. (1997). A mathematical model of circulation in the presence of the bidirectional cavopulmonary anastomosis in children with a univentricular heart. Med Eng. Phys..

[114] Pennati G., Fumero R. (2000). Scaling approach to study the changes through the gestation of human fetal cardiac and circulatory behaviors. Ann. Biomed. Eng..

[115] Shimizu S., Une D., Kawada T., Hayama Y., Kamiya A., Shishido T., Sugimachi M. (2018). Lumped parameter model for hemodynamic simulation of congenital heart diseases. J. Physiol. Sci..

[116] Duanmu Z., Yin M., Fan X., Yang X., Luo X. (2018). A patient-specific lumped-parameter model of coronary circulation. Sci. Rep..

[117] Spilker R.L., Taylor C.A. (2010). Tuning multidomain hemodynamic simulations to match physiological measurements. Ann. Biomed. Eng..

[118] Ménigault E., Vieyres P., Lepoivre B., Durand A., Pourcelot L., Berson M. (1997). Fetal heart modelling based on a pressure-volume relationship. Med Biol. Eng. Comput..

[119] Garber L., Khodaei S., Keshavarz-Motamed Z. (2022). The critical role of lumped parameter models in patient-specific cardiovascular simulations. Arch. Comput. Methods Eng..

[120] Moin P. (2010). Fundamentals of Engineering Numerical Analysis.

[121] Pennati G., Bellotti M., Fumero R. (1997). Mathematical modelling of the human foetal cardiovascular system based on Doppler ultrasound data. Med Eng. Phys..

[122] Lingman G., Maršal K. (1986). Fetal central blood circulation in the third trimester of normal pregnancy-a longitudinal study. I. Aortic and umbilical blood flow. Early Hum. Dev..

[123] Guettouche A., Papapanayotou C., Cherruault Y., Azancot-Benisty A., Challier J. (1993). Optimization and resolution algorithm of the human fetal blood circulation model. Math. Comput. Model..

[124] Sutton M., Groves A., MacNeill A., Sharland G., Allan L. (1994). Assessment of changes in blood flow through the lungs and foramen ovale in the normal human fetus with gestational age: A prospective Doppler echocardiographic study. Heart.

[125] Capper W., Myers L. Gestational age dependency of umbilical flow waveforms: An investigation using a lumped parameter model. Proceedings of the 22nd Annual International Conference of the IEEE Engineering in Medicine and Biology Society (Cat. No.00CH37143).

[126] Kenny J.F., Plappert T., Doubilet P., Saltzman D.H., Cartier M., Zollars L., Leatherman G., St John Sutton M. (1986). Changes in intracardiac blood flow velocities and right and left ventricular stroke volumes with gestational age in the normal human fetus: A prospective Doppler echocardiographic study. Circulation.

[127] Ferrazzi E., Gementi P., Bellotti M., Rodolfi M., Della Peruta S., Barbera A., Pardi G. (1991). Doppler velocimetry: Critical analysis of umbilical, cerebral and aortic reference values. Eur. J. Obstet. Gynecol. Reprod. Biol..

[128] Hecher K., Campbell S., Snijders R., Nicolaides K. (1994). Reference ranges for fetal venous and atrioventricular blood flow parameters. Ultrasound Obstet. Gynecol. Off. J. Int. Soc. Ultrasound Obstet. Gynecol..

[129] Pennati G., Corno C., Costantino M.L., Bellotti M. (2003). Umbilical flow distribution to the liver and the ductus venosus in human fetuses during gestation: An anatomy-based mathematical modeling. Med. Eng. Phys..

[130] van den Wijngaard J.P.H.M., Westerhof B.E., Faber D.J., Ramsay M.M., Westerhof N., van Gemert M.J.C. (2006). Abnormal arterial flows by a distributed model of the fetal circulation. Am. J. Physiol.-Regul. Integr. Comp. Physiol..

[131] Bellotti M., Pennati G., Pardi G., Fumero R. (1998). Dilatation of the ductus venosus in human fetuses: Ultrasonographic evidence and mathematical modeling. Am. J. Physiol.-Heart Circ. Physiol..

[132] Grigioni M., Carotti A., Daniele C., D’Avenio G., Morbiducci U., Di Benedetto G., Albanese S., Di Donato R., Barbaro V. (2001). A mathematical model of the fetal cardiovascular system based on genetic algorithms as identification technique. Int. J. Artif. Organs.

[133] Struijk P.C., Mathews V.J., Loupas T., Stewart P.A., Clark E.B., Steegers E.A.P., Wladimiroff J.W. (2008). Blood pressure estimation in the human fetal descending aorta. Ultrasound Obstet. Gynecol..

[134] Luria O., Bar J., Shalev J., Kovo M., Golan A., Barnea O. (2014). Inverse solution of the fetal-circulation model based on ultrasound Doppler measurements. Cardiovasc. Eng. Technol..

[135] Garcia-Cañadilla P., Crispi F., Cruz-Lemini M., Triunfo S., Nadal A., Valenzuela-Alcaraz B., Rudenick P.A., Gratacos E., Bijnens B.H. (2015). Patient-specific estimates of vascular and placental properties in growth-restricted fetuses based on a model of the fetal circulation. Placenta.

[136] Kulkarni A., Garcia-Cañadilla P., Khan A., Lorenzo J.M., Beckerman K., Valenzuela-Alcaraz B., Cruz-Lemini M., Gomez O., Gratacos E., Crispi F. (2018). Remodeling of the cardiovascular circulation in fetuses of mothers with diabetes: A fetal computational model analysis. Placenta.

[137] Hooper S., Pas A., Lang J., Van Vonderen J., Roehr C., Kluckow M., Gill A., Wallace E., Polglase G. (2015). Cardiovascular transition at birth: A physiological sequence. Pediatr. Res..

[138] Sá-Couto C.D., Andriessen P., Van Meurs W.L., Ayres-de Campos D., Sá-Couto P.M. (2010). A model for educational simulation of hemodynamic transitions at birth. Pediatr. Res..

[139] Yigit B., Tutsak E., Yıldırım C., Hutchon D., Pekkan K. (2019). Transitional fetal hemodynamics and gas exchange in premature postpartum adaptation: Immediate vs. delayed cord clamping. Matern. Heal. Neonatol. Perinatol..

[140] Rasanen J., Wood D.C., Weiner S., Ludomirski A., Huhta J.C. (1996). Role of the pulmonary circulation in the distribution of human fetal cardiac output during the second half of pregnancy. Circulation.

[141] Wiputra H., Lai C.Q., Lim G.L., Heng J.J.W., Guo L., Soomar S.M., Leo H.L., Biwas A., Mattar C.N.Z., Yap C.H. (2016). Fluid mechanics of human fetal right ventricles from image-based computational fluid dynamics using 4D clinical ultrasound scans. Am. J. Physiol.-Heart Circ. Physiol..

[142] Lai C.Q., Lim G.L., Jamil M., Mattar C.N.Z., Biswas A., Yap C.H. (2016). Fluid mechanics of blood flow in human fetal left ventricles based on patient-specific 4D ultrasound scans. Biomech. Model. Mechanobiol..

[143] Groenenberg I., Stijnen T., Wladimiroff J. (1990). Blood flow velocity waveforms in the fetal cardiac outflow tract as a measure of fetal well-being in intrauterine growth retardation. Pediatr. Res..

[144] Arvidsson P.M., Kovács S.J., Töger J., Borgquist R., Heiberg E., Carlsson M., Arheden H. (2016). Vortex ring behavior provides the epigenetic blueprint for the human heart. Sci. Rep..

[145] Sahn D.J., Lange L.W., Allen H.D., Goldberg S.J., Anderson C., Giles H., Haber K. (1980). Quantitative real-time cross-sectional echocardiography in the developing normal humam fetus and newborn. Circulation.

[146] Salman H.E., Kamal R.Y., Yalcin H.C. (2021). Numerical Investigation of the Fetal Left Heart Hemodynamics During Gestational Stages. Front. Physiol..

[147] Salman H.E., Kamal R.Y., Hijazi Z.M., Yalcin H.C. (2022). Hemodynamic and Structural Comparison of Human Fetal Heart Development Between Normally Growing and Hypoplastic Left Heart Syndrome-Diagnosed Hearts. Front. Physiol..

[148] Wiputra H., Lim G.L., Chia D.A.K., Mattar C.N.Z., Biswas A., Yap C.H. (2016). Methods for fluid dynamics simulations of human fetal cardiac chambers based on patient-specific 4D ultrasound scans. J. Biomech. Sci. Eng..

[149] Wiputra H., Lim G.L., Chua K.C., Nivetha R., Soomar S.M., Biwas A., Mattar C.N.Z., Leo H.L., Yap C.H. (2017). Peristaltic-like motion of the human fetal right ventricle and its effects on fluid dynamics and energy dynamics. Ann. Biomed. Eng..

[150] Vasudevan V., Wiputra H., Yap C.H. (2019). Torsional motion of the left ventricle does not affect ventricular fluid dynamics of both foetal and adult hearts. J. Biomech..

[151] Zebhi B., Wiputra H., Howley L., Cuneo B., Park D., Hoffman H., Gilbert L., Yap C.H., Bark D. (2020). Right ventricle in hypoplastic left heart syndrome exhibits altered hemodynamics in the human fetus. J. Biomech..

[152] Chen Z., Zhou Y., Wang J., Liu X., Ge S., He Y. (2017). Modeling of coarctation of aorta in human fetuses using 3D/4D fetal echocardiography and computational fluid dynamics. Echocardiography.

[153] Chen Z., Zhao H., Zhao Y., Han J., Yang X., Throckmorton A., Wei Z., Ge S., He Y. (2022). Retrograde flow in aortic isthmus in normal and fetal heart disease by principal component analysis and computational fluid dynamics. Echocardiography.

[154] Dean W.R. (1927). Note on the motion of fluid in a curved pipe. Lond. Edinb. Dublin Philos. Mag. J. Sci..

[155] Germano M. (1982). On the effect of torsion on a helical pipe flow. J. Fluid Mech..

[156] Kaplan A.D., Jaffa A.J., Timor I.E., Elad D. (2010). Hemodynamic analysis of arterial blood flow in the coiled umbilical cord. Reprod. Sci..

[157] Shah R.G., Girardi T., Merz G., Necaise P., Salafia C.M. (2017). Hemodynamic analysis of blood flow in umbilical artery using computational modeling. Placenta.

[158] Saw S.N., Poh Y.W., Chia D., Biswas A., Mattar C.N.Z., Yap C.H. (2018). Characterization of the hemodynamic wall shear stresses in human umbilical vessels from normal and intrauterine growth restricted pregnancies. Biomech. Model. Mechanobiol..

[159] Wen J., Tang J., Ran S., Ho H. (2020). Computational modelling for the spiral flow in umbilical arteries with different systole/diastole flow velocity ratios. Med. Eng. Phys..

[160] Saw S.N., Dawn C., Biswas A., Mattar C.N.Z., Yap C.H. (2017). Characterization of the in vivo wall shear stress environment of human fetus umbilical arteries and veins. Biomech. Model. Mechanobiol..

[161] Wilke D., Denier J., Khong T., Mattner T. (2018). Pressure and flow in the umbilical cord. J. Biomech..

[162] Tejada-Martínez A.E., Borberg C.J., Venugopal R., Carballo C., Moreno W.A., Quintero R.A. (2011). Computational fluid dynamic analysis of flow velocity waveform notching in umbilical arteries. Am. J. Physiol.-Regul. Integr. Comp. Physiol..

[163] Kasiteropoulou D., Topalidou A., Downe S. (2020). A computational fluid dynamics modelling of maternal-fetal heat exchange and blood flow in the umbilical cord. PLoS ONE.

[164] Chato J.C. (1980). Heat transfer to blood vessels. J. Biomed. Eng..

[165] Kolios M.C., Sherar M., Hunt J. (1995). Large blood vessel cooling in heated tissues: A numerical study. Phys. Med. Biol..

[166] Amare R., Hodneland E., Roberts J.A., Bahadori A.A., Eckels S. (2022). Modeling a 3-D multiscale blood-flow and heat-transfer framework for realistic vascular systems. Sci. Rep..

[167] Liu X., Chen X., Zhang Y., Xie J., Jia X., Deng T., Zheng Y., Davood T., Majid Z. (2022). The thermal behavior of blood flow in the arteries with various radii and various stenosis angles using non-Newtonian Sisko model. Alex. Eng. J..

[168] Jiji L.M. (2009). Heat Transfer in Living Tissue. Heat Conduction: Third Edition.

[169] Pennati G., Redaelli A., Bellotti M., Ferrazzi E. (1996). Computational analysis of the ductus venosus fluid dynamics based on Doppler measurements. Ultrasound Med. Biol..

[170] Leinan P.R., Degroote J., Kiserud T., Skallerud B., Vierendeels J., Hellevik L.R. (2013). Velocity profiles in the human ductus venosus: A numerical fluid structure interaction study. Biomech. Model. Mechanobiol..

[171] Acharya G., Kiserud T. (1999). Pulsations of the ductus venosus blood velocity and diameter are more pronounced at the outlet than at the inlet. Eur. J. Obstet. Gynecol. Reprod. Biol..

[172] Pennati G., Bellotti M., Ferrazzi E., Rigano S., Garberi A. (1997). Hemodynamic changes across the human ductus venosus: A comparison between clinical findings and mathematical calculations. Ultrasound Obstet. Gynecol..

[173] Leinan P.R., Kiserud T., Hellevik L.R. (2013). Human ductus venosus velocity profiles in the first trimester. Cardiovasc. Eng. Technol..

[174] Pennati G., Bellotti M., Ferrazzi E., Bozzo M., Pardi G., Fumero R. (1998). Blood Flow Through the Ductus Venosus in Human Fetus: Calculation Using Doppler Velocimetry and Computational Findings. Ultrasound Med. Biol..

[175] Haugen G., Kiserud T., Godfrey K., Crozier S., Hanson M. (2004). Portal and umbilical venous blood supply to the liver in the human fetus near term. Ultrasound Obstet. Gynecol..

[176] Poelmann R.E., Gittenberger-de Groot A.C. (2018). Hemodynamics in Cardiac Development. J. Cardiovasc. Dev. Dis..

[177] Kung E., Kahn A.M., Burns J.C., Marsden A. (2014). In vitro validation of patient-specific hemodynamic simulations in coronary aneurysms caused by Kawasaki disease. Cardiovasc. Eng. Technol..

[178] Roldán-Alzate A., García-Rodríguez S., Anagnostopoulos P.V., Srinivasan S., Wieben O., François C.J. (2015). Hemodynamic study of TCPC using in vivo and in vitro 4D Flow MRI and numerical simulation. J. Biomech..

[179] Sincomb S., Coenen W., Sánchez A.L., Lasheras J.C. (2020). A model for the oscillatory flow in the cerebral aqueduct. J. Fluid Mech..

[180] Kung E.O., Les A.S., Figueroa C.A., Medina F., Arcaute K., Wicker R.B., McConnell M.V., Taylor C.A. (2011). In vitro validation of finite element analysis of blood flow in deformable models. Ann. Biomed. Eng..

[181] Marquis A.D., Arnold A., Dean-Bernhoft C., Carlson B.E., Olufsen M.S. (2018). Practical identifiability and uncertainty quantification of a pulsatile cardiovascular model. Math. Biosci..

[182] Bjørdalsbakke N.L., Sturdy J.T., Hose D.R., Hellevik L.R. (2022). Parameter estimation for closed-loop lumped parameter models of the systemic circulation using synthetic data. Math. Biosci..

[183] Schrauben E.M., Darby J.R., Saini B.S., Holman S.L., Lock M.C., Perumal S.R., Seed M., Morrison J.L., Macgowan C.K. (2020). Technique for comprehensive fetal hepatic blood flow assessment in sheep using 4D flow MRI. J. Physiol..

